# Magnificines A and B, Antimicrobial Marine Alkaloids Featuring a Tetrahydrooxazolo[3,2-a]azepine-2,5(*3H,6H*)-dione Backbone from the Red Sea Sponge *Negombata magnifica*

**DOI:** 10.3390/md19040214

**Published:** 2021-04-12

**Authors:** Diaa T. A. Youssef, Hani Z. Asfour, Grégory Genta-Jouve, Lamiaa A. Shaala

**Affiliations:** 1 Department of Natural Products, Faculty of Pharmacy, King Abdulaziz University, Jeddah 21589, Saudi Arabia; 2 Department of Medical Parasitology, Faculty of Medicine, Princess Al-Jawhara Center of Excellence in Research of Hereditary Disorders, King Abdulaziz University, Jeddah 21589, Saudi Arabia; hasfour@kau.edu.sa; 3 UMR 8038 CiTCoM, Faculté de Pharmacie de Paris, Université Paris Descartes, Avenue de l’observatoire, 75006 Paris, France; gregory.genta-jouve@parisdescartes.fr; 4 Molecules of Communication and Adaptation of Microorganisms (UMR 7245), National Museum of Natural History, CNRS, 75231 Paris, France; 5 Natural Products Unit, King Fahd Medical Research Center, King Abdulaziz University, Jeddah 21589, Saudi Arabia; 6 Department of Medical Laboratory Sciences, Faculty of Applied Medical Sciences, King Abdulaziz University, Jeddah 21589, Saudi Arabia; 7 Suez Canal University Hospital, Suez Canal University, Ismailia 41522, Egypt

**Keywords:** Red Sea sponge, *Negombata magnifica*, marine alkaloids, β-ionone, magnificines A and B, (±)-negombaionone, latrunculin B and 16-*epi*-latrunculin B, antimicrobial activity, *E. coli*, cell line growth inhibition, HeLa cells

## Abstract

Investigation of the Red Sea sponge *Negombata magnifica* gave two novel alkaloids, magnificines A and B (**1** and **2**) and a new β-ionone derivative, (±)-negombaionone (**3**), together with the known latrunculin B (**4**) and 16-*epi*-latrunculin B (**5**). The analysis of the NMR and HRESIMS spectra supported the planar structures and the relative configurations of the compounds. The absolute configurations of magnificines A and B were determined by the analysis of the predicted and experimental ECD spectra. Magnificines A and B possess a previously unreported tetrahydrooxazolo[3,2-*a*]azepine-2,5(*3H,6H*)-dione backbone and represent the first natural compounds in this class. (±)-Negombaionone is the first β-ionone of a sponge origin. Compounds **1**-**3** displayed selective activity against *Escherichia coli* in a disk diffusion assay with inhibition zones up to 22 mm at a concentration of 50 µg/disc and with MIC values down to 8.0 µM. Latrunculin B and 16-*epi*-latrunculin B inhibited the growth of HeLa cells with IC_50_ values down to 1.4 µM.

## 1. Introduction

Sponges belonging to the genus *Negombata* (formerly *Latrunculia*) [1] (pp. 698–699) are characterized by diverse secondary metabolites of different classes including macrolides (latrunculins) [2,3,4,5,6,7,8,9], pyrroloiminoquinone alkaloids (discorhabdins) [10,11,12,13,14,15,16,17], terpene peroxides [18,19], cyclic 2-oxecanone glycosides [20], diterpenes [21], ceramides [22,23], and peptides [24,25,26]. Reported latrunculins displayed anticancer, antiviral, antibiotic, antiangiogenic, antimigratory, and microfilament-disrupting activities [2,3,4,5,6,7,8,9]. Pyrroloiminoquinone alkaloids exhibited antimicrobial, immunomodulatory, caspase inhibition, antiviral, feeding deterrence, and antimalarial properties and present potent inhibition potential of mammalian topoisomerase II in vivo [10,11,12,13,14,15,16,17]. Additional pharmacological activities for other chemical entities identified from the genus *Negombata* include cytotoxicity [18,19,21], antifeeding [20], antiepileptic, and anti-inflammatory [22,23], potent inotropic effects and inhibition of the cardiac Na/Ca exchanger [24,25,26].

As a part of our growing interest to discover biologically active leads from marine resources [27,28,29], the organic extract of the sponge *Negombata magnifica* was examined. Two alkaloids, magnificines A and B (**1** and **2**), with a previously unreported tetrahydrooxazolo[3,2-*a*]azepine-2,5(*3H,6H*)-dione skeleton were purified. In addition, a new β-ionone derivative, (±)-negombaionone (**3**), with the previously reported latrunculin B (**4**) [2] and 16-*epi*-latrunculin B (**5**) [5] were obtained. Structural determinations of **1**-**5** were accomplished by HRESIMS and NMR spectral analyses.

## 2. Results and Discussion

### 2.1. Purification of **1**-**5**

**Figure 1:**
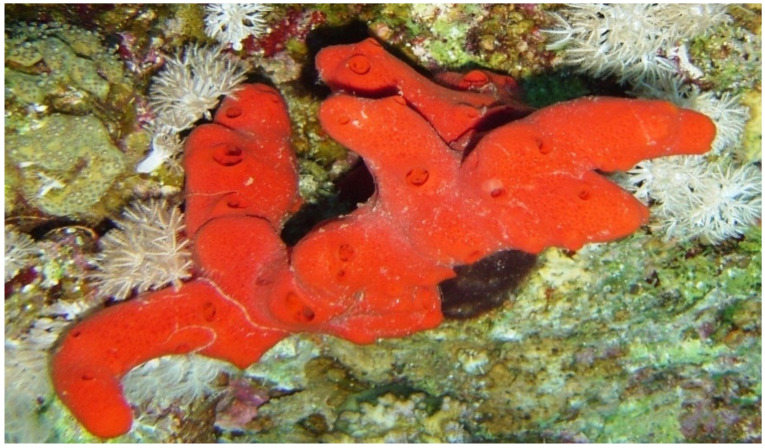
Underwater photograph of the Red Sea *Negombata magnifica*.

Fractionation of the methanolic extract of *N. magnifica* [30] (Figure 1) using partition (on silica gel), size exclusion (Sephadex LH 20), and purification of active fractions on HPLC afforded **1**-**5**.

### 2.2. Structure of Magnificine A (1)

**Figure 2:**
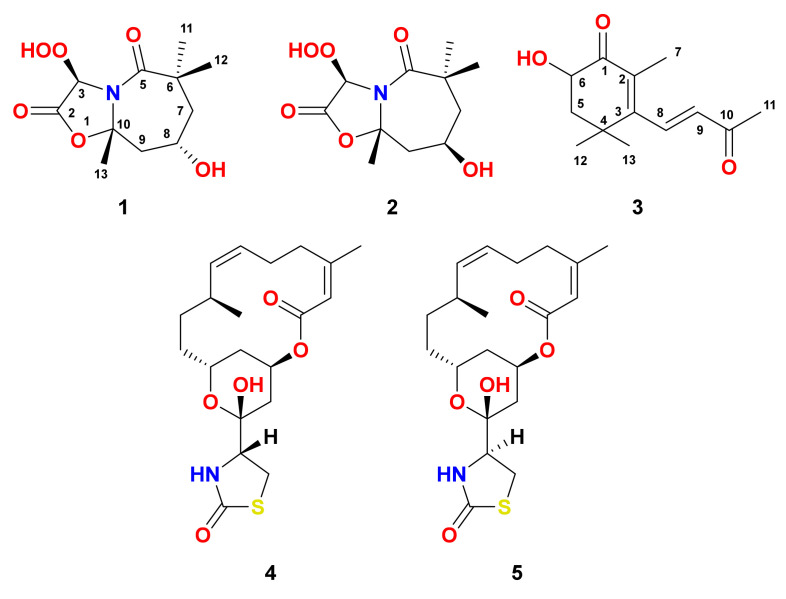
Chemical structures of **1**-**5**.

**Figure 3:**
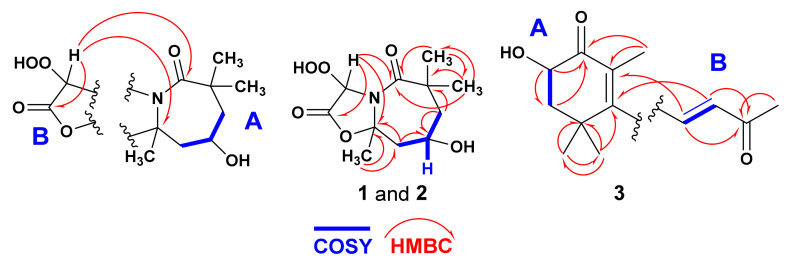
Subunits of **1** and **3**, and COSY and HMBC of **1**-**3**.

Magnificine A (**1**) (Figure 2) obtained as an optically active ([α]$\begin{array}{c} 25\\ D \end{array}$ = + 70°) oil. The chemical structure of **1** was determined from interpretation of its MS and NMR spectra (Figures S1–S10). The HRESIMS data (*m*/*z* = 282.0961, C_11_H_17_NNaO_6_, [M + Na]^+^) supported molecular formula C_11_H_17_NO_6_, suggesting four degrees of unsaturation. Its ^13^C NMR spectrum and HSQC experiment exhibited 11 signals including four quaternary carbons, two oxygenated methines, two methylenes and three methyls (Figure 2 and Table 1). The combined ^1^H NMR spectrum and COSY experiment supported the existence of a single ^1^H-^1^H coupling system from H_2_-7 to H_2_-9 (CH_2_-7–CH-8–CH_2_-9) (Figure 3). Beside the geminal coupling between the protons at C-7 (δ_H_ 2.03 and 1.33, ^2^*J*_7a,7b_ = 11.5 Hz), vicinal couplings from H-7a (^3^*J*_7a,8_ = 4.2 Hz) and H-7b (^3^*J*_7b,8_ = 11.5 Hz) to the oxygenated methine H-8 (δ_H_ 4.13, tt, *J* = 11.5, 4.2 Hz) were observed. Furthermore, H-8 exhibited additional vicinal (^3^*J*_HH_) couplings to H-9a (δ_H_ 2.54, ddd, *J* = 11.5, 4.2, 1.8 Hz) and H-9b (δ_H_ 1.51, t, *J* = 11.5 Hz) completing the coupling system.

The ^13^C NMR resonances at δ_C_ 49.8 (CH_2_, C-7), 65.1 (CH, C-8), and 47.9 (CH_2_, C-9) are correlated to the protons at δ_H_ 2.03/1.33 (H-7a and H-7b), 4.13 (H-8), and 2.54/1.51 (H-9a and H-9b) in the HSQC experiment, supporting the assignment of these signals and the placement of OH group at C-8. The interruption of the spin-coupling system of H_2_-7–H-8–H_2_-9 on both sides suggests the quaternary nature of C-6 and C-10. The substituents at C-6 and C-10, and the existence of an amidic carbonyl (δ_C_ 180.7, C-5) were confirmed from the HMBC of H_3_-11/C-6, H_3_-12/C-6, H_3_-11/C-7, H_3_-12/C-7, H_3_-11/C-5, H_3_-12/C-5, H_3_-13/C-9, H_3_-13/C-10, H_2_-7/C-5, and H_2_-9/C-10 (Table 1 and Figure 3), completing the structure of the seven-membered ring (Fragment A). The remaining elements of C_2_H_2_O_4_ (Fragment B) displayed two signals in the ^1^H and ^13^C NMR spectra at δ_H/C_ 171.5 (qC, C-2) and 5.72/113.3 (CH, s, H-3/C-3) corresponding to a carbonyl of a lactone moiety and an oxygenated methine. The downfield chemical shift of C-10 at δ_C_ 86.4 (qC) supported its attachment to the heteroatoms (O and N) of the lactone and amide functionalities. The HMBC of H-3/C-10, H_2_-9/C-10 and H_3_-10/C-10 supported this assignment. The appearance of the NMR signals of H-3/C-3 at δ_H/C_ 5.72/113.3 supported the attachment C-3 to the N atom of the amidic group of the seven-membered ring as well as the presence of the remaining elements (OOH) at C-3, completing the molecular formula of **1**. The attachment of the two fragments (five- and seven-membered rings) of **1** through *N*-4–C-10 was supported from ^3^*J*_CH_ HMBC from H-3 (δ_H_ 5.72) to C-5 (δ_C_ 180.7) and C-10 (δ_C_ 86.4) (Figure 3), completing the planar structure of **1**.

**Figure 4:**
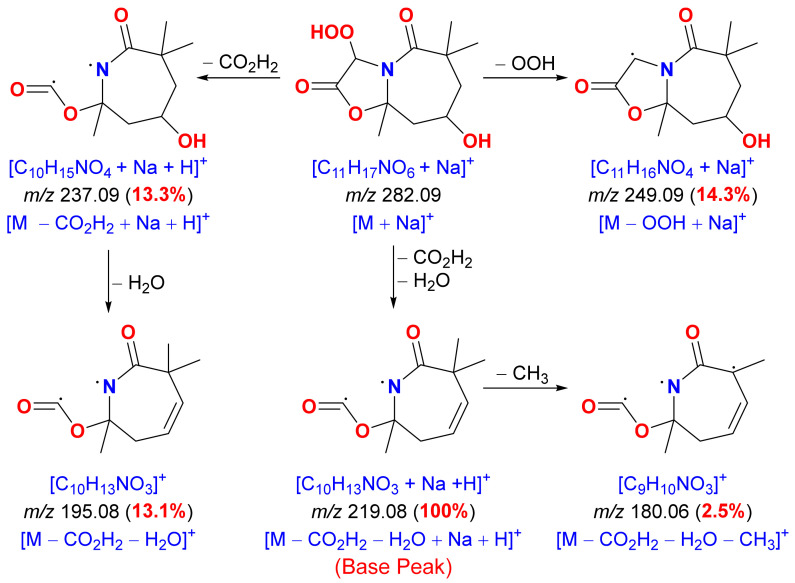
Significant MS ion fragments of magnificine A (**1**).

The planar structure of **1** as well as the substitution on both subunits of **1** were confirmed again from the MS ion peaks at *m*/*z* 249.09 (14.3%), 237.09 (13.3%), 219.08 (100%, base peak), 195.08 (13.1%) and 180.06 (2.3%) (Figure 4) in the ESIMS. The ion peak at *m*/*z* 249.09 results from the loss of OOH moiety [M − OOH + Na]^+^ from the parent ion peak at *m*/*z* 282.09 [M + Na]^+^. Consecutive loss of CO_2_H_2_ and H_2_O fragments from both sides of the compound results in an ion peak at *m*/*z* 219.08 (base peak) [M − CO_2_H_2_ − H_2_O + Na + H]^+^. Further loss of CH_3_ group from the base peak gives a minor ion peak at *m*/*z* 180.06 [M − CO_2_H_2_ − H_2_O − CH_3_]^+^. The loss of CO_2_H_2_ from the five-membered ring gives an ion peak at *m*/*z* 237.09 [M − CO_2_H_2_ + Na + H]^+^, which further lose H_2_O from the seven-membered ring resulting in an ion peak at *m*/*z* 195.08 [M − CO_2_H_2_ − H_2_O]^+^ (Figure 4).

**Figure 5:**
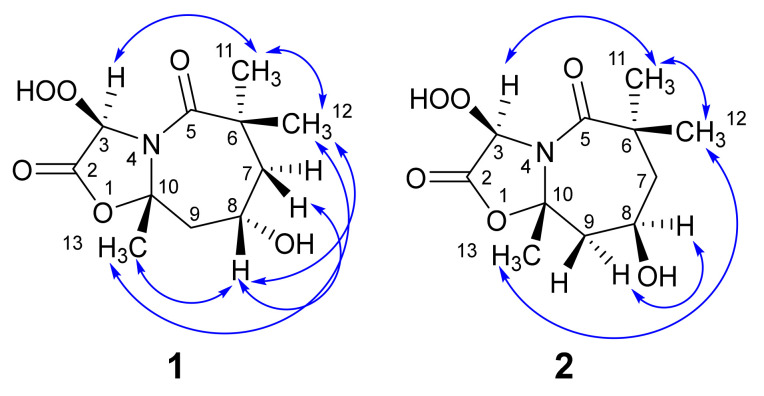
Significant NOESY correlations of **1** and **2**.

The strong NOESY correlations between H-8 and H_3_-12, and between H-8 and H_3_-13 confirm the same relative configurations of such functionalities (Figure 5). Further, the NOESY between H-3 and H_3_-11 supported the same configuration as well as the opposite configuration to H-8, H_3_-12 and H_3_-13 (Table 1 and Figure 5).

**Figure 6:**
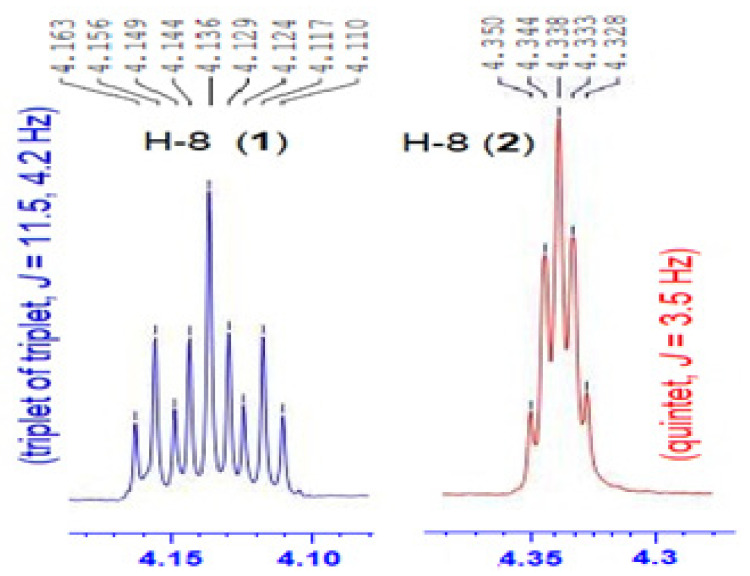
Multiplicity of H-8 in **1** (blue) and **2** (red).

**Figure 7:**
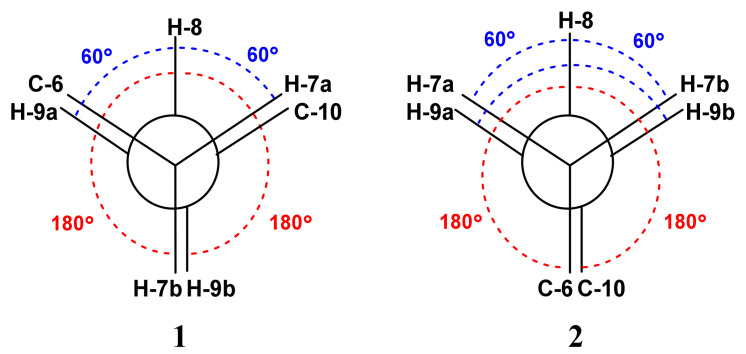
Anticipated dihedral angles between H-8 and adjacent methylenic protons (H-7a, H-7b, H-9a and H-9b) in **1** and **2**.

The magnitude of the vicinal ^3^*J*_HH_ values between H-8 (tt, *J* = 11.5, 4.2 Hz) (Figure 6) and H-7b (δ_H_ = 1.33, ^3^*J*_8,7b_ = 11.5 Hz), and between H-8 and H-9b (δ_H_ = 1.51, ^3^*J*_8,9b_ = 11.5 Hz) suggests similar dihedral angles of 180° [31] between H-8 and both H-7b and H-9b (Figure 7). On the contrary, the values of the vicinal ^3^*J*_HH_ values between H-8 and H-7a (δ_H_ = 2.03, ^3^*J*_8,7a_ = 4.2 Hz) and between H-8 and H-9a (δ_H_ = 2.54, ^3^*J*_8,9a_ = 4.2 Hz) suggest similar dihedral angles of 60° [31] between H-8 and H-7a, and between H-8 and H-9a (Figure 7).

**Figure 8:**
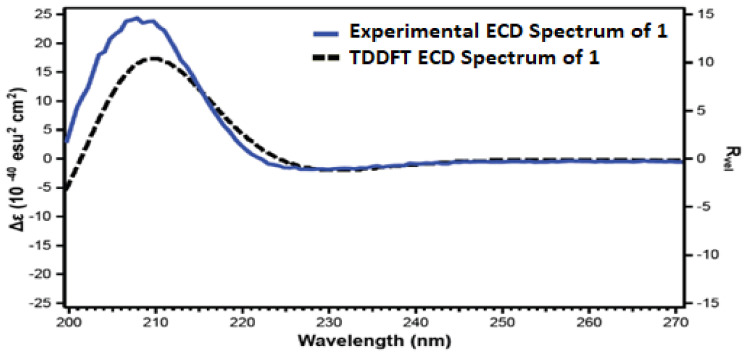
Experimental and calculated ECD spectra of **1**.

The absolute configurations at the stereogenic carbons C-3, C-8 and C-10 of **1** were confirmed from comparison of the experimental and TDDFT-predicted ECD spectra (Figure 8). A good agreement between both ECD spectra was noticed. The sign of the unique Cotton Effect (CE) due to the n→π* transition of the lactone enabled the assignment of the configurations at the stereogenic centers as 3*R*,8*S*,10*S*. Accordingly, **1** was assigned as (3*R*,8*S*,9a*S*)-3-hydroperoxy-8-hydroxy-6,6,9a-trimethyltetrahydrooxazolo[3,2-a]azepine-2,5(3*H*,6*H*)-dione and named magnificine A.

### 2.3. Structure of Magnificine B (2)

Magnificine B (**2**) (Figure 2), an optically active ([α]$\begin{array}{c} 25\\ D \end{array}$ = −65°) compound, with molecular formula of C_11_H_17_NO_6_ (*m*/*z* = 282.0961, C_11_H_17_NNaO_6_, [M + Na]^+^). Interpretation of the MS and NMR spectra of **2** (Figures S11–S19) supported its structure determination. Inspection of the NMR spectra of **1** and **2** (Table 1 and Table 2) showed high similarity between the ^1^H and ^13^C chemical shifts, suggesting similar planar structure of both compounds. The appearance of oxymethine H-8 in **2** as a quintet (δ_H_ 4.33, quin., ^3^*J* = 3.5 Hz) instead of triplet of triplet (δ_H_ 4.13, tt, ^3^*J* = 11.5 and 4.2 Hz) in **1** suggested an opposite configuration of the OH moiety at C-8.

The NOESY cross-peaks between H-8 and H_3_-13, H-8 and H-7b, H-8 and H-9a, and between H_3_-12 and H_3_-13 supported the similar configuration of these moieties (Table 2 and Figure 5). Further, a NOESY between H_3_-11 and H-3 supported the same configuration (Figure 5). Additionally, the same ^3^*J* value of 3.5 Hz between H-8 (quin., *J* = 3.5 Hz) (Figure 6) and the four methylenic protons (H-7a, H-7b, H-9a and H-9b) proposed similar dihedral angles of 60° [31] between H-8 and these protons (H-7a, H-7b, H-9a and H-9b) (Figure 7).

**Figure 9:**
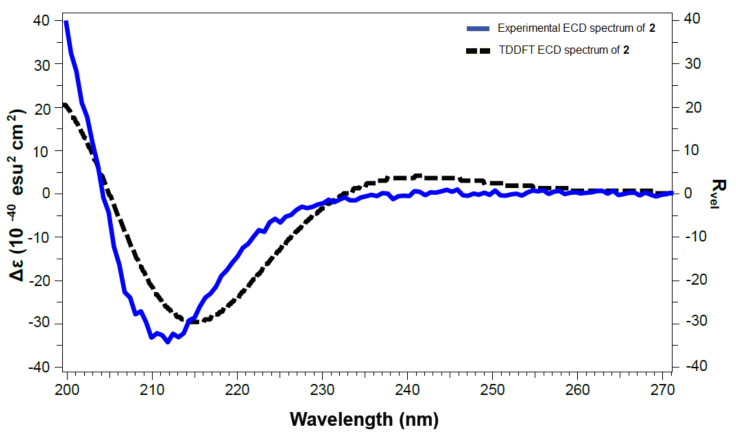
Experimental and calculated ECD spectra of **2**.

The absolute configurations at C-3, C-8, and C-10 of **2** were determined by comparison between the predicted and the experimental ECD spectra (Figure 9). In comparison to compound **1**, the sign of the unique CE was inverted in **2**, suggesting opposite configuration at C-8 (Table 2 and Figure 5). Thus, the configurations at C-3, C-8 and C-10 was confirmed to be 3*R*,8*R*,10*S*. Thus, **2** was assigned as (3*R*,8*R*,9a*S*)-3-hydroperoxy-8-hydroxy-6,6,9a-trimethyltetrahydrooxazolo[3,2-a]azepine-2,5(3*H*,6*H*)-dione and named magnificine B.

Magnificines A and B represent the first natural compounds with a tetrahydrooxazolo[3,2-*a*]azepine-2,5(*3H,6H*)-dione backbone. Their occurrence highlights exceptional biosynthetic and chemical biotransformation capabilities in marine sponges.

### 2.4. Structure of (±)-Negombaionone (3)

Compound **3** (Figure 2) was purified as an optically inactive ([α]$\begin{array}{c} 25\\ D \end{array}$ = −65°) solid. The positive HRESIMS (*m*/*z* 245.1157, C_13_H_18_NaO_3_ [M + Na]^+^) supported the molecular formula of C_13_H_18_O_3_. The analyses of its NMR and MS spectra (Figures S20–S26) proved its chemical structure. Its ^13^C NMR spectrum revealed 13 resonances divided into four methyls, one methylene, two olefinic methines, and five quaternary carbons, as supported by the HSQC experiment (Table 3). The interpretation of ^1^H, ^13^C, COSY, HSQC and HMBC of **2** supported the assignment of two subunits in **3** as 2,3,4,6-terasubstituted cyclohex-2-en-1-one (subunit A) and buta-3-en-2-one (subunit B) linked together via C-3/C-8 (Figure 2). The ^1^H and ^13^C NMR resonances at δ_H/C_ 200.2 (qC, C-1), 128.8 (qC, C-2), 158.6 (qC, C-3), 36.7 (qC, C-4), 2.21 (1H, dd), 1.86(1H, t)/45.1 (CH_2_, H_2_-5/C-5), 4.37 (1H, dd)/69.3 (CH, C-6) (Table 3) supported the presence of subunit A. Vicinal couplings between H-6 and the geminal-coupled protons at C-5 (H-5a and H-5b) were observed. Further, HMBC of H-6/C-1, H_2_-5/C-1, H_3_-7/C-1, H_3_-7/C-2, H-8/C-2, H_3_-7/C-3, H-9/C-3, H_3_-12/C-3, H_3_-13/C-3, H_2_-5/C-4, H_3_-12/C-4, H_3_-13/C-4, H-6/C-5, H_3_-12/C-5, H_3_-13/C-5, H_2_-5/C-6, and H_3_-12/C-6 (Figure 3 and Table 3) confirmed the assignment of subunit A. Similarly, subunit B was assigned from the ^1^H/^13^C signals at δ_H/C_ 7.20 (1H, dd)/139.4 (CH, C-8), 6.22 (1H, d)/134.1 (CH, C-9), 197.3 (qC, C-10) and 2.36 (3H, s)/28.2 (CH_3_, C-11). The *E* configuration at C-8/C-9 was supported by a ^3^*J* value of 16.5 Hz between H-8 and H-9. The HMBC cross-peaks of H-8/C-9, H-8/C-10, H-9/C-10 and H_3_-11/C-10 (Figure 3 and Table 3) completed the assignment of subunit B. The connection of subunits A and B via C-3/C-8 was supported from HMBC of H-8/C-2 and H-9/C-3, completing the planar structure of **3**.

The racemic nature of **3** was confirmed from the absence of any optical activity ([α]$\begin{array}{c} 25\\ D \end{array}$ = 0°) as well as from the absence of any CE in the experimental ECD spectrum. Thus, **3** was confirmed to be a racemic mixture and was assigned as (±)-(*E*)-6-hydroxy-2,4,4-trimethyl-3-(3-oxobut-1-en-1-yl)cyclohex-2-en-1-one and named (±)-negombaionone.

**Figure 10:**
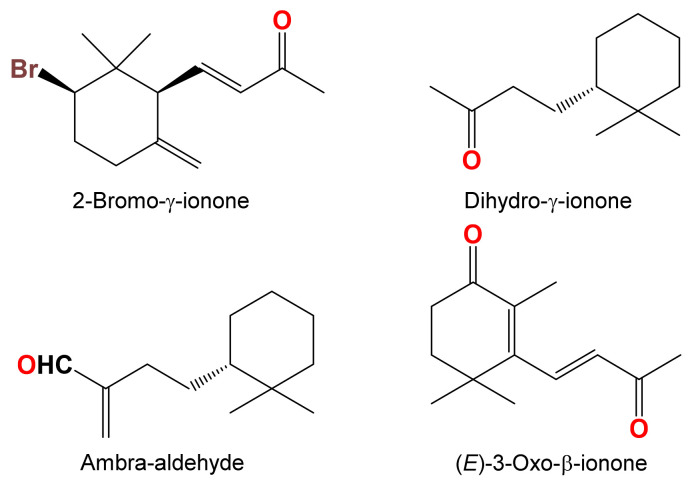
Representative examples of marine-derived ionones [32,33,34].

Ionones represent a rare class of secondary metabolites in marine organisms. Only four candidates including 2-bromo-γ-ionone [32], (*E*)-3-oxo-β-ionone [33], dihydro-γ-ionone and ambra-aldehyde [34] are of marine origin (Figure 10). (±)-Negombaionone represents the first β-ionone of a sponge origin.

Compounds **4** and **5** were identified by an interpretation of their NMR (Figures S27–30) and MS data and by comparison with the data in the literature [30,31]. Accordingly, compounds **4** and **5** were characterized as latrunculin B [2] and 16-*epi*-latrunculin B [5], respectively.

Compounds **1**-**3** were investigated for their antimicrobial activities against three pathogens. Compounds **1**-**3** displayed selective activity against *E. coli* (ATCC 25922) at a concentration of 50 µg/disc in a disk diffusion assay with inhibition zones of 22, 20 and 20 mm, respectively. Further, **1**-**3** exhibited equal MIC values of 8, 8 and 8 µM, respectively, against *E. coli* in a microdilution assay. The compounds were inactive against *S. aureus* (ATCC 25923) and *C. albicans* (ATCC 14053). These results suggest selective activity of **1**-**3** against *E. coli*. These findings support the importance of marine sponges as a vigorous foundation of antimicrobial secondary metabolites and the potential of future development of **1**-**3** as antimicrobial leads.

In an MTT assay, latrunculin B (**4**) and 16-*epi*-latrunculin B (**5**) displayed growth inhibition of HeLa cells with IC_50_ values of 1.4 and 3.9 µM, respectively, suggesting the selectivity of **4** and **5** against HeLa cells.

## 3. Materials and Methods

### 3.1. General Experimental Procedures

The optical rotations and spectral data of **1**-**5** including UV, ECD, NMR and MS are acquired as previously reported [27,28,29].

### 3.2. Biological Materials

The brick-red sponge *Negombata magnifica* KellyBorges and Vacelet (order Poecilosclerida, suborder Mycalina, family Podospongiidae) [30] was collected as branched fingerlike strips by hands using SCUBA at depths of 20–25 from the Red Sea coast (N 021°39′17.5″, E 038°52′26.3″). A specimen with number KSA-119 was reserved at King Abdulaziz University.

### 3.3. Purification of Compounds **1**-**5**

The freeze-dried material (85 g) was soaked in MeOH overnight (3 × 650 mL). Combined extracts were partitioned on a VLC silica column using hexane-EtOAc-MeOH gradients. The fraction eluted with 80% EtOAc in hexane was subjected twice to partition on Sephadex LH-20 using CH_2_Cl_2_-MeOH (1:1) to give four subfractions (Fr. A-D). The antibacterial fraction (Fr. B, 19 mg) (inhibition zone = 10 mm against *E. coli*, at 100 µg/disc) was purified on HPLC (C18, AR II Cosmosil 250 × 10 mm, 5 μm, Waters) using H_2_O-MeCN (60:40) at 3 mL/min to give compounds **1** (1.6 mg) (*t*_R_ = 13.0 min), **2** (1.2 mg) (*t*_R_ = 14.0 min) and **3** (2.7 mg) (*t*_R_ = 15.0 min). Similarly, the cytotoxic fraction (Fr. C, 24 mg) (IC_50_ = 7 µg/mL against HeLa cells) was purified on HPLC (C18, Gemini^®^ 5 μm, 250 × 0.64 mm, Phenomenex) using H_2_O-MeCN (40:60) at 1 mL/min to give **4** (17 mg) (*t*_R_ = 8.8 min) (17 mg) and **5** (3.5 mg) (*t*_R_ = 9.5 min).

### 3.4. Spectral Data of the Compounds

Magnificine A (**1**): Yellow oil; [α]$\begin{array}{c} 25\\ D \end{array}$ 70° (*c* 0.1, MeOH); UV (MeOH) *λ*_max_ (log *ε*): 201 (2.89), 274 (2.69) nm; ECD (MeOH) [Δε]_212 nm_ +22.01; HRESIMS *m*/*z* 282.0961 (calcd for C_11_H_17_O_6_NNa, [M + Na]^+^, 282.0953).

Magnificine B (**2**): Yellow oil; [α]$\begin{array}{c} 25\\ D \end{array}$ −65° (*c* 0.1, MeOH); UV (MeOH) *λ*_max_ (log *ε*): 201 (2.80), 274 (2.66) nm; ECD (MeOH) [Δε]_219 nm_ −6.00; HRESIMS *m*/*z* 282.0961 (calcd for C_11_H_17_O_6_NNa, [M + Na]^+^, 282.0953).

(±)-Negombaionone (**3**): Off-white solid; m.p.: 138 °C; [α]$\begin{array}{c} 25\\ D \end{array}$ 0° (*c* 0.1, MeOH); UV (MeOH) *λ*_max_ (log *ε*): 205 (2.75), 274 (2.34) nm; IR (film): ν_max_ 3520, 1681, 1663, 1607, 1079, 984 cm^−1^; HRESIMS *m*/*z* 245.1157 (calcd for C_13_H_18_O_3_Na, [M + H]^+^, 245.1153).

### 3.5. Computational Details

The calculations of the DFT were carried out using Gaussian 16 [35] and as previously reported [28].

### 3.6. Disk Diffusion Assay

The evaluation of the antimicrobial activities of **1**-**3** against *E. coli*, *S. aureus* and *C. albicans* were carried out using a disk diffusion assay at 50 µg/disc as reported earlier [36,37,38].

### 3.7. MIC of the Compounds

The evaluation of the MIC values of compounds **1**-**3** against *E. coli* was performed using a macrodilution method as reported before [36,39].

### 3.8. MTT Assay

The evaluation of the growth inhibition activities of compounds **4** and **5** against HeLa cells (ATCC CCL-2) was carried out as previously reported using an MTT assay [27,40].

## 4. Conclusions

Sponges of the genus *Negombata* continue to provide profound chemical entities with previously unknown motifs. Two novel alkaloids, magnificines A (**1**) and B (**2**), together with a new β-ionone derivative, (±)-negombaionone (**3**), and the known latrunculin B (**4**) and 16-*epi*-latrunculin B (**5**), were purified from the antimicrobial and cytotoxic fractions of the sponge *N. magnifica*. The structural characterizations of **1**-**5** were supported by analyses of their NMR and MS data. Absolute configurations of **1** and **2** were established by comparison of the predicted and experimental ECD spectra. Magnificines A and B possess an unprecedented tetrahydrooxazolo[3,2-*a*]azepine-2,5(*3H,6H*)-dione backbone. Magnificines A and B and (±)-negombaionone displayed selective activity towards *E. coli* without any effect on *S. aureus* and *C. albicans.* On the other hand, latrunculin B and 16-*epi*-latrunculin B displayed significant growth inhibition activities towards HeLa cells. The current results suggest that **1**-**3** could be a foundation for the development of novel antibacterial leads.

## Figures and Tables

**Table 1:** NMR data of **1** (600 MHz for ^1^H and 150 for ^13^C, CDCl_3_).

No.	δ_C_ (mult.)	δ_H_ [mult., *J* (Hz)]	HMBC	NOESY
2	171.5, qC		H-3	
3	113.3, CH	5.72 (s)		H_3_-11
5	180.7, qC		H-3, H_2_-7, H_3_-11, H_3_-12	
6	35.0, qC		H_3_-11, H_3_-12, H_2_-7	
7a	49.8, CH_2_	2.03 (ddd, 11.5, 4.2, 2.4)	H_3_-11, H_3_-12, H_2_-9	
7b		1.33 (t, 11.5)		
8	65.1, CH	4.13 (tt, 11.5, 4.2)	H_2_-7, H_2_-9	H-7b, H_3_-12, H_3_-13
9a	47.9, CH_2_	2.54 (ddd, 11.5, 4.2, 1.8)	H_2_-7, H_3_-13	
9b		1.51 (t, 11.5)		
10	86.4, qC		H_3_-13, H-3, H_2_-9	
11	29.9, CH_3_	1.31 (s)	H_3_-12	H-3
12	25.1, CH_3_	1.27 (s)	H_3_-11	H-8, H_3_-13
13	25.6, CH_3_	1.59 (s)		H-8, H_3_-12

**Table 2:** NMR data of **2** (600 MHz for ^1^H and 150 MHz for ^13^C, CDCl_3_).

No.	δ_C_ (mult.)	δ_H_ [mult., *J* (Hz)]	HMBC	NOESY
2	171.9, qC		H-3	
3	112.9, CH	5.70 (s)		H_3_-11
5	182.3, qC		H-3, H_3_-11, H_3_-12	
6	35.9, qC		H-8, H_3_-11, H_3_-12, H_2_-7, H-3	
7a	47.3, CH_2_	2.47 (td, 14.5, 3.5, 3.5)	H_3_-11, H_3_-12	
7b		1.79 (dd, 14.5, 3.5)		H-8
8	66.8, CH	4.33 (quin, 3.5)		H-7b, H-9a, H-9b
9a	45.6, CH_2_	1.97 (td, 14.5, 3.5, 3.5)	H_3_-13	H-8
9b		1.53 (dd, 14.5, 3.5)		H-8
10	86.6, qC		H-3, H-8, H_3_-13	
11	30.6, CH_3_	1.27 (s)	H_3_-12	H-3
12	26.4, CH_3_	1.47 (s)	H_3_-11	H_3_-13
13	27.0, CH3	1.78 (s)		H_3_-12

**Table 3:** NMR data of **3** (600 MHz for ^1^H, 150 MHz for ^13^C, CDCl_3_).

No.	δ_C_ (mult.)	δ_H_ [mult., *J* (Hz)]	HMBC
1	200.2, qC		H-6, H_2_-5, H_3_-7
2	128.8, qC		H_3_-7, H-8
3	158.6, qC		H_3_-7, H-9, H_3_-12, H_3_-13
4	36.7, qC		H_2_-5, H_3_-12, H_3_-13
5a 5b	45.1, CH_2_	2.21 (dd, 14.0, 6.0) 1.86 (t, 14.0)	H-6, H_3_-12, H_3_-13
6	69.3, CH	4.37 (dd, 14.0, 6.0)	H_2_-5, H_3_-12
7	13.5, CH_3_	1.88 (d, 0.6)	
8	139.4, CH	7.20 (dd, 16.5, 0.6)	
9	134.1, CH	6.22 (d, 16.5)	H-8
10	197.3, qC		H-8, H-9, H_3_-11
11	28.2, CH_3_	2.36 (s)	H_3_-12
12	30.3, CH_3_	1.17 (s)	H_3_-13
13	25.7, CH_3_	1.35 (s)	H_3_-12, H_2_-5

## Supplementary Materials

The following are available online at https://www.mdpi.com/article/10.3390/md19040214/s1, Figures S1–S10: 1HNMR, 13C NMR, DEPT, COSY, HSQC, and HMBC, NOESY, LRESIMS and HRESIMS spectra of magnificine A (1), Figures S11–S19: 1HNMR, 13C NMR, DEPT, COSY, HSQC, and HMBC, NOESY and HRESIMS spectra of magnificine B (2), Figures S20–S26: 1HNMR, 13C NMR, DEPT, COSY, HSQC, HMBC and HRESIMS spectra of (±)-negombaionone (3), Figures S27 and S28: 1H and 13C NMR spectra of latrunculin B (4), Figures S29 and S30: 1H and 13C NMR spectra of 16-epi-latrunculin B (**5**).
