# Supplementary material for: Magnificines A and B, Antimicrobial Marine Alkaloids Featuring a Tetrahydrooxazolo[3,2-a]azepine-2,5(3H,6H)-dione Backbone from the Red Sea Sponge Negombata magnifica
Source: Mar Drugs. 2021 Apr 12;19(4):214. doi: 10.3390/md19040214 (PMC8068863; doi:10.3390/md19040214)

Supporting Information

Figure S1. ^{1}H NMR spectrum of magnificine A (**1**) ············ 1
Figure S2. Expansion of ^{1}H NMR spectrum of magnificine A (**1**) ············ 1
Figure S3. ^{13}C NMR spectrum of magnificine A (**1**) ············ 2
Figure S4. DEPT spectrum of magnificine A (**1**) ············ 2
Figure S5. ^{1}H-^{1}H COSY spectrum of magnificine A (**1**) ············ 3
Figure S6. Multiplicity-edited HSQC spectrum of magnificine A (1) ············ 3
Figure S7. ^{1}H-^{13}C HMBC spectrum of magnificine A (**1**) ············ 4
Figure S8. NOESY spectrum of magnificine A (**1**) ············ 4
Figure S9. HRESIMS spectrum of magnificine A (**1**) ············ 5
Figure S10. LRESIMS spectrum and MS fragment ion peaks of magnificine A (**1**) ······· 6
Figure S11. ^{1}H NMR spectrum of magnificine B (**2**) ············ 7
Figure S12. Expansion of ^{1}H NMR spectrum of magnificine B (**2**) ············ 7
Figure S13. ^{13}C NMR spectrum of magnificine B (**2**) ············ 8
Figure S14. DEPT spectrum of magnificine B (**2**) ············ 8
Figure S15. ^{1}H-^{1}H COSY spectrum of magnificine B (**2**) ············ 9
Figure S16. Multiplicity-edited HSQC spectrum of magnificine B (**2**) ············ 9
Figure S17. ^{1}H-^{13}C HMBC spectrum of magnificine B (**2**) ············ 10
Figure S18. NOESY spectrum of magnificine B (**2**) ············ 10
Figure S19. HRESIMS spectrum of magnificine B (**2**) ············ 11
Figure S20. ^{1}H NMR spectrum of (±)-negombaionone (**3**) ············ 12
Figure S21. ^{13}C NMR spectrum of (±)-negombaionone (**3**) ············ 12
Figure S22. DEPT spectrum of (±)-negombaionone (**3**) ············ 12
Figure S23. ^{1}H-^{1}H COSY spectrum of (±)-negombaionone (**3**) ············ 14
Figure S24. Multiplicity-edited HSQC spectrum of (±)-negombaionone (**3**) ············ 14
Figure S25. ^{1}H-^{13}C HMBC spectrum of (±)-negombaionone (**3**) ············ 15
Figure S26. HRESIMS spectrum of (±)-negombaionone (**3**) ············ 15
Figure S27. ^{1}H NMR spectrum of latrunculin B (**4**) ············ 16
Figure S28. ^{13}C NMR spectrum of latrunculin B (**4**) ············ 16
Figure S29. ^{1}H NMR spectrum of 16-epilatrunculin B (**5**) ············ 17
Figure S30. ^{13}C NMR spectrum of 16-epilatrunculin B (**5**) ············ 17

Figure S1. ^{1}H NMR spectrum of magnificine A (**1**) ($CDCl_3$).

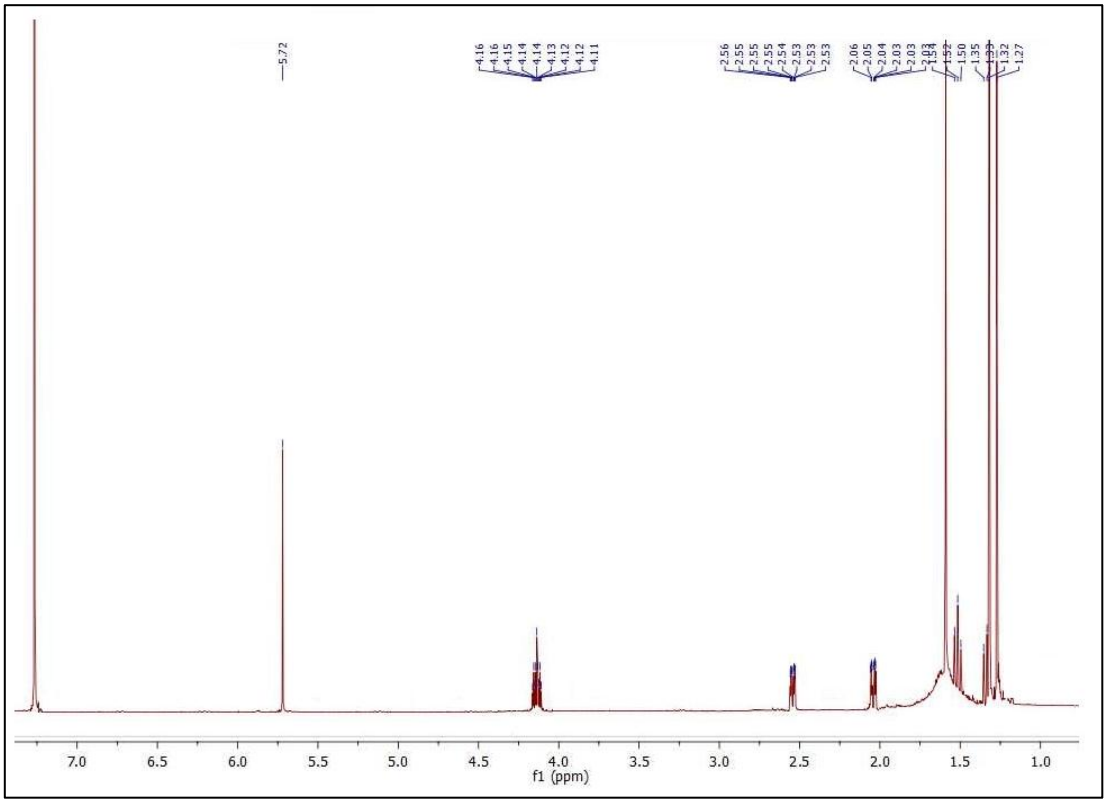

Figure S2. Expansion of ^{1}H NMR spectrum of magnificine A (**1**) ($CDCl_3$).

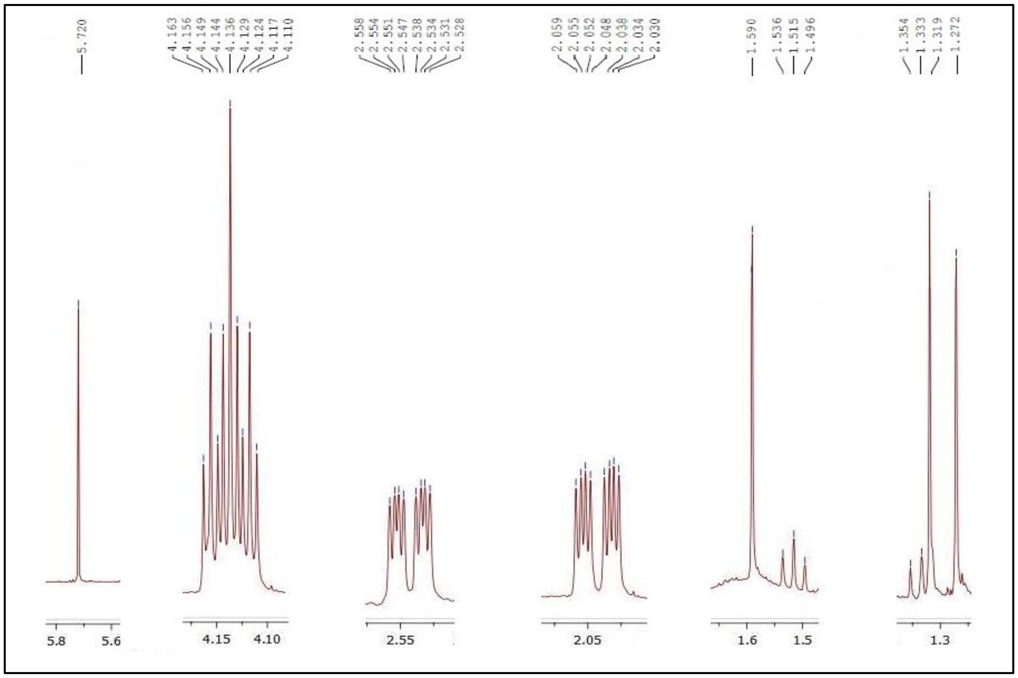

Figure S3. ^{13}C NMR spectrum of magnificine A (**1**) (CDCl$_3$).

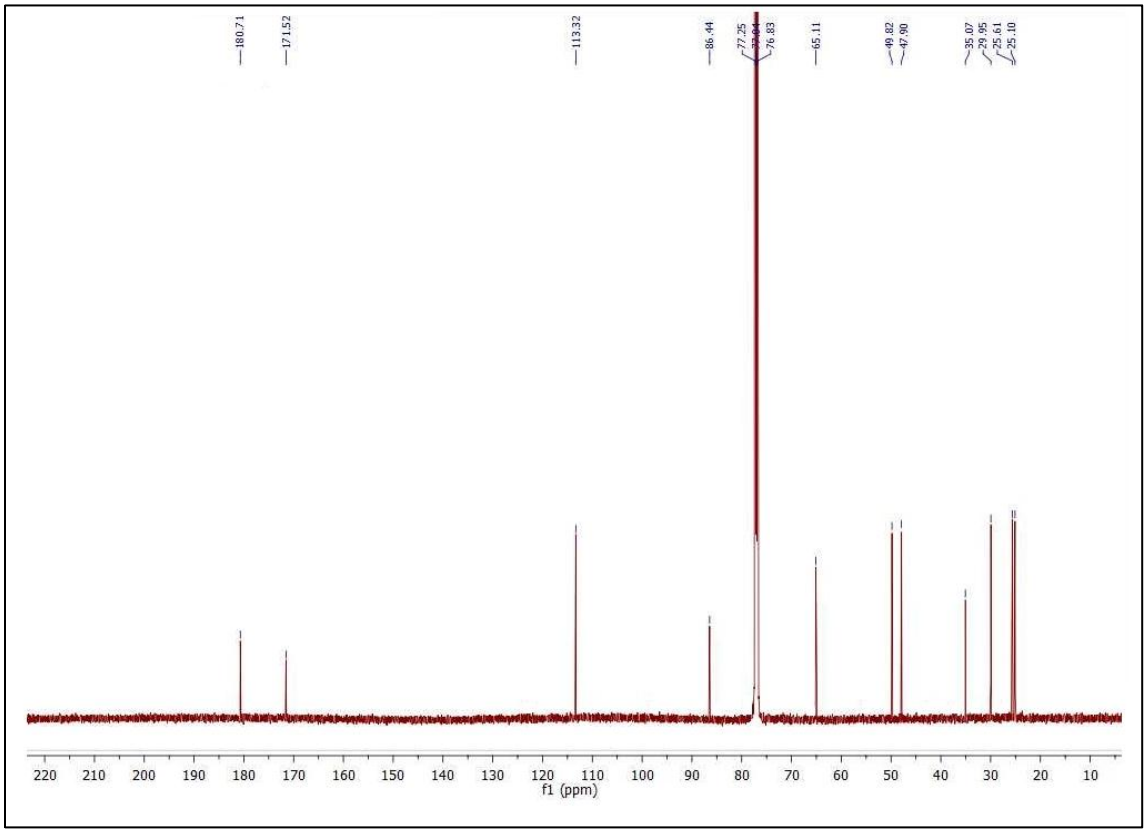

Figure S4. DEPT spectrum of magnificine A (**1**) (CDCl$_3$).

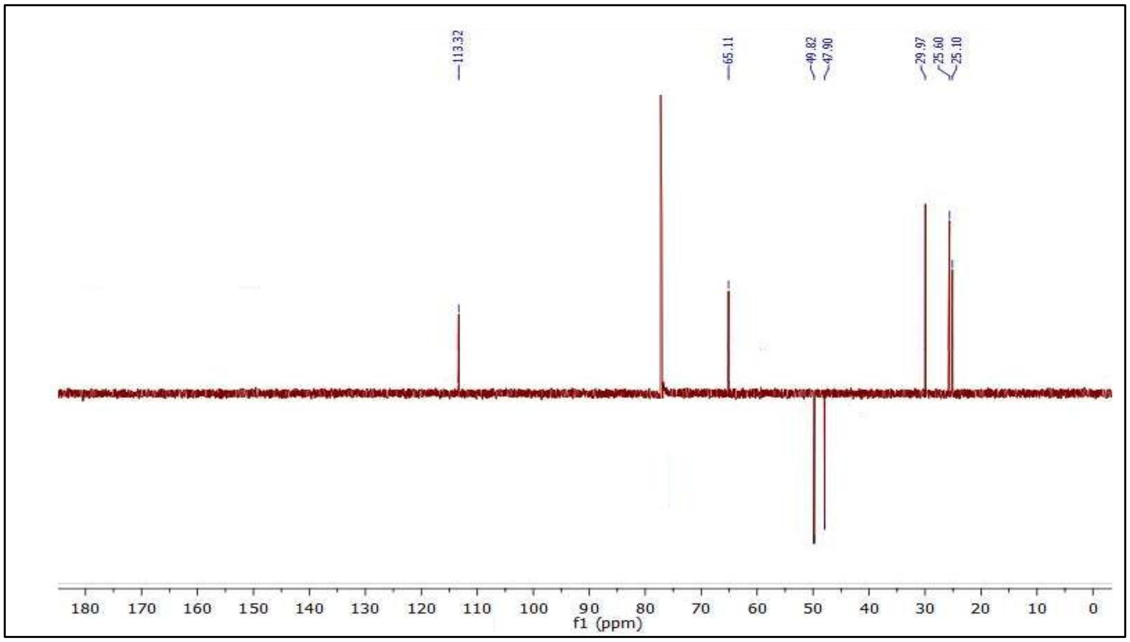

Figure S5. ^{1}H-^{1}H COSY spectrum of magnificine A (**1**) ($CDCl_3$).

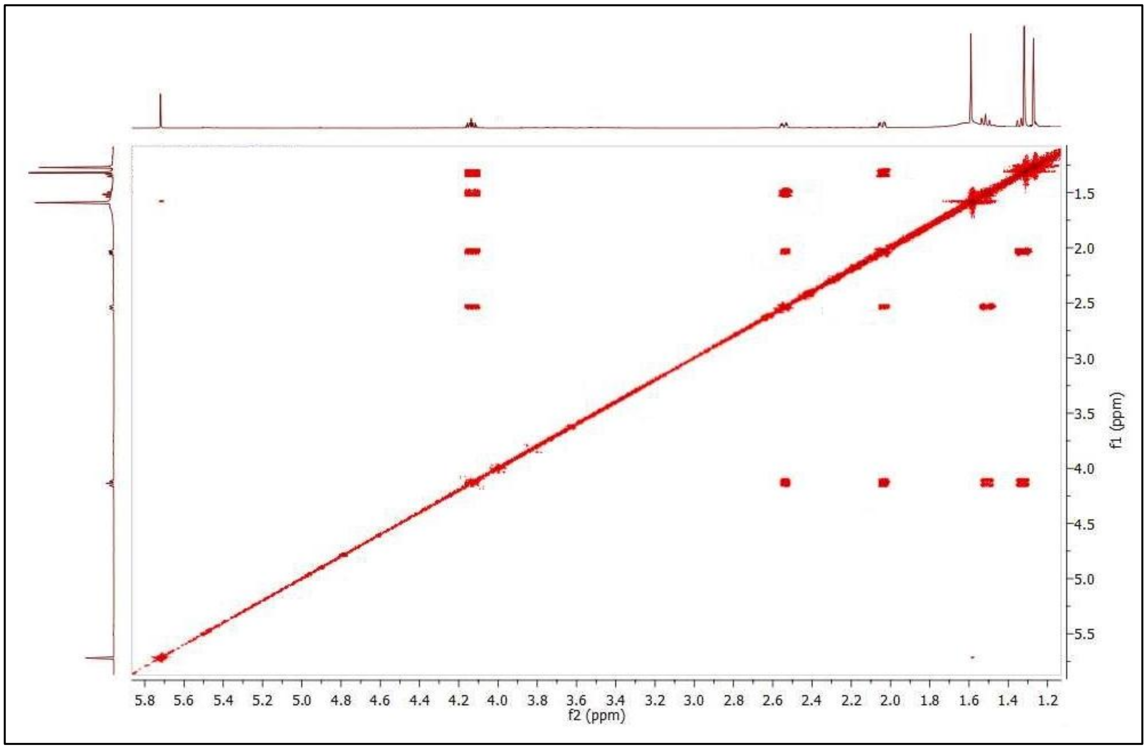

Figure S6. Multiplicity-edited HSQC spectrum of magnificine A (**1**) ($CDCl_3$).

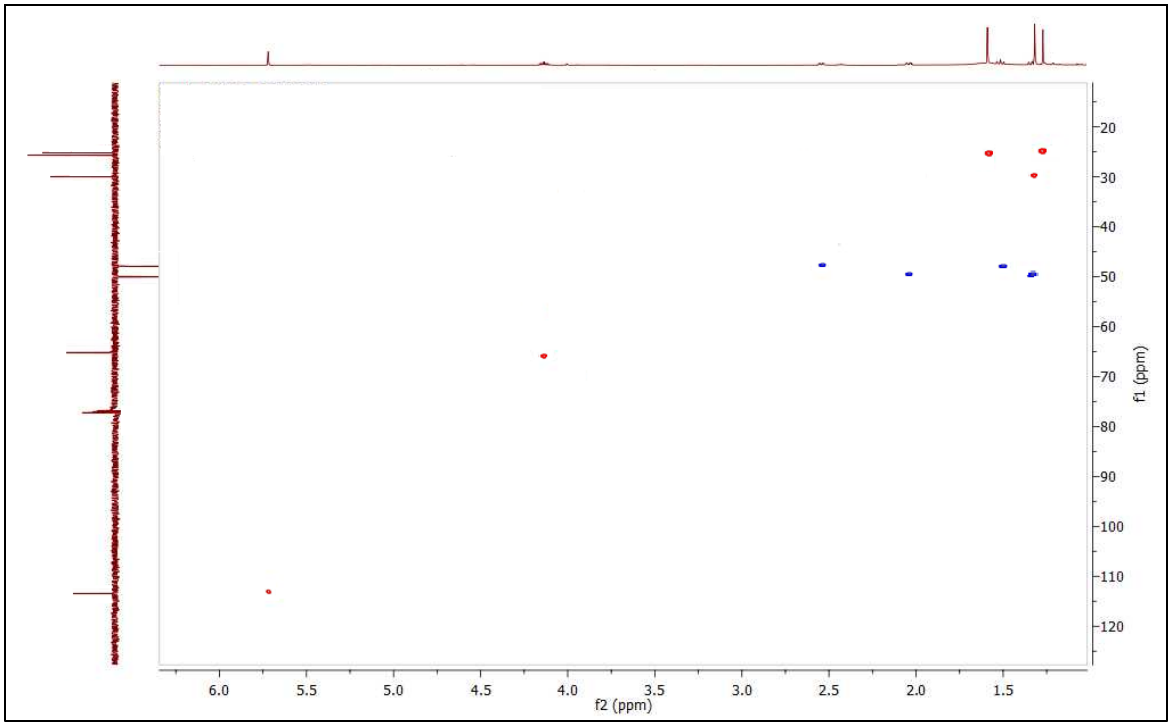

Figure S7. ^{1}H-^{13}C HMBC spectrum of magnificine A (**1**) ($CDCl_3$).

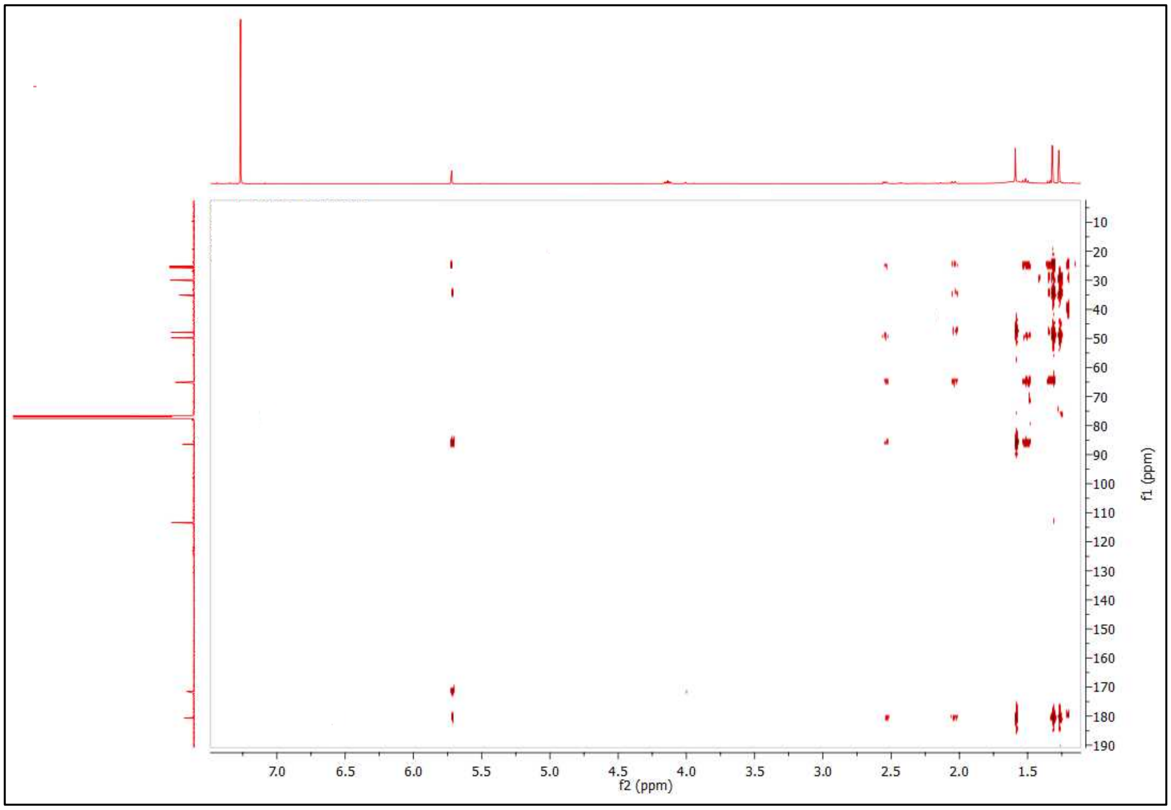

Figure S8. ^{1}H-^{1}H NOESY spectrum of magnificine A (**1**) ($CDCl_3$).

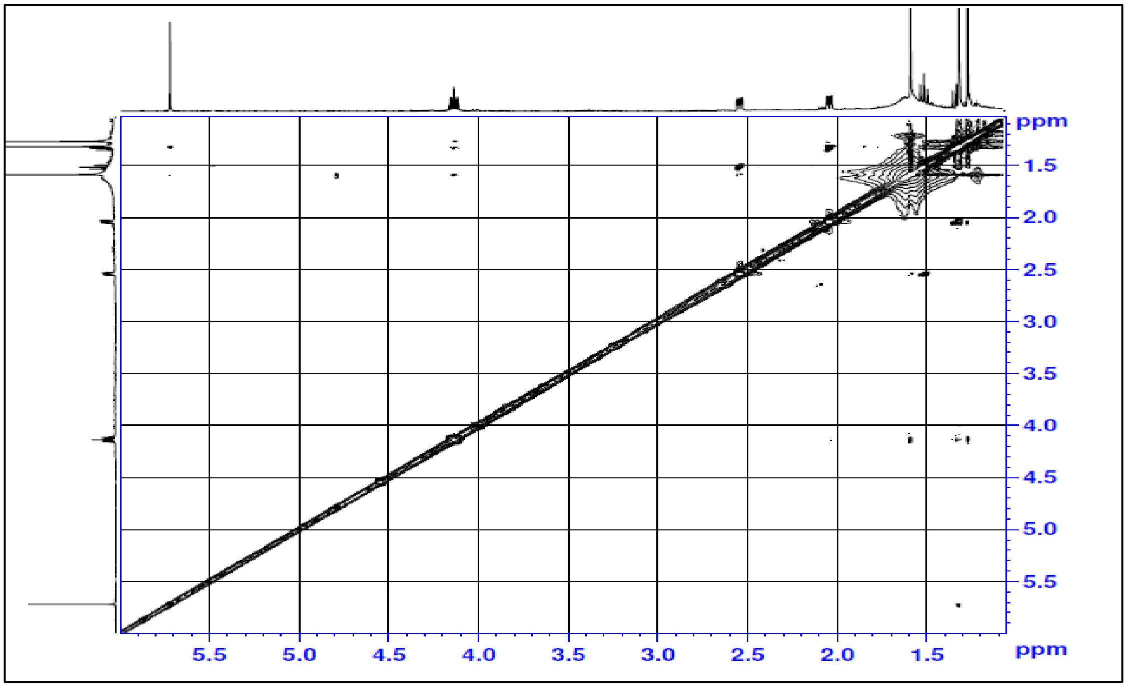

Figure S9. HRESIMS spectrum of magnificine A (**1**).

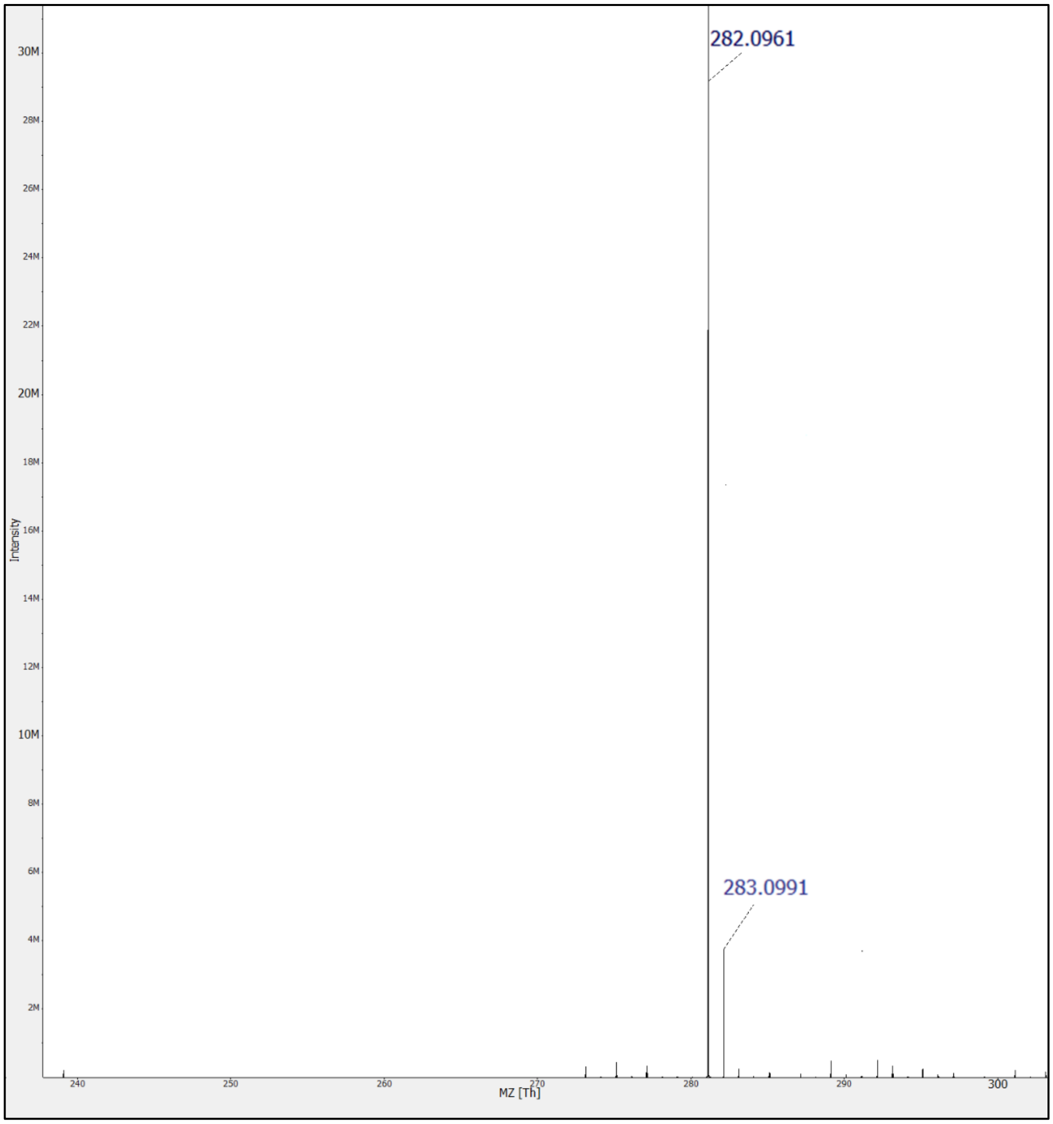

Figure S10. LRESIMS spectrum and MS fragment ion peaks of magnificine A (**1**).

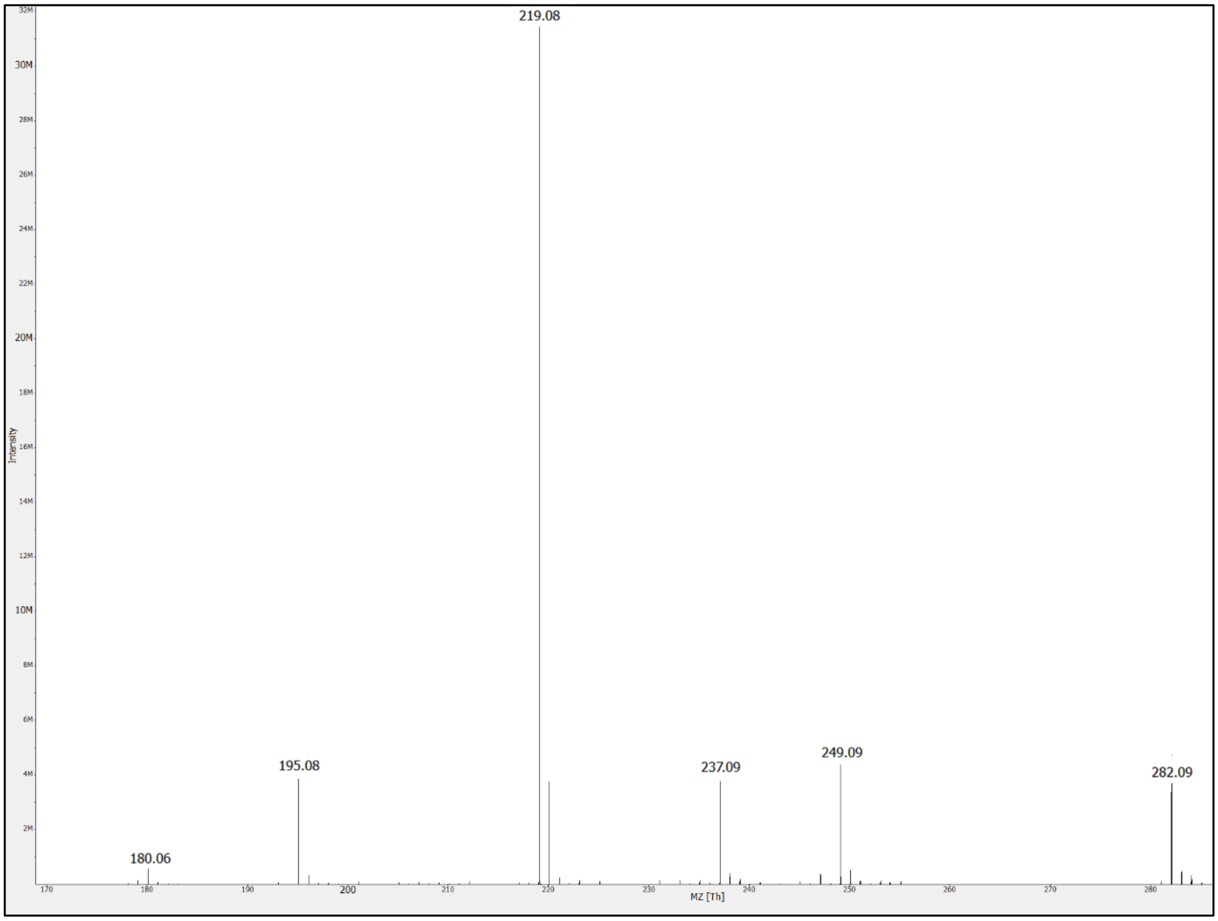

Figure S11. ^{1}H NMR spectrum of magnificine B (**2**) ($CDCl_3$).

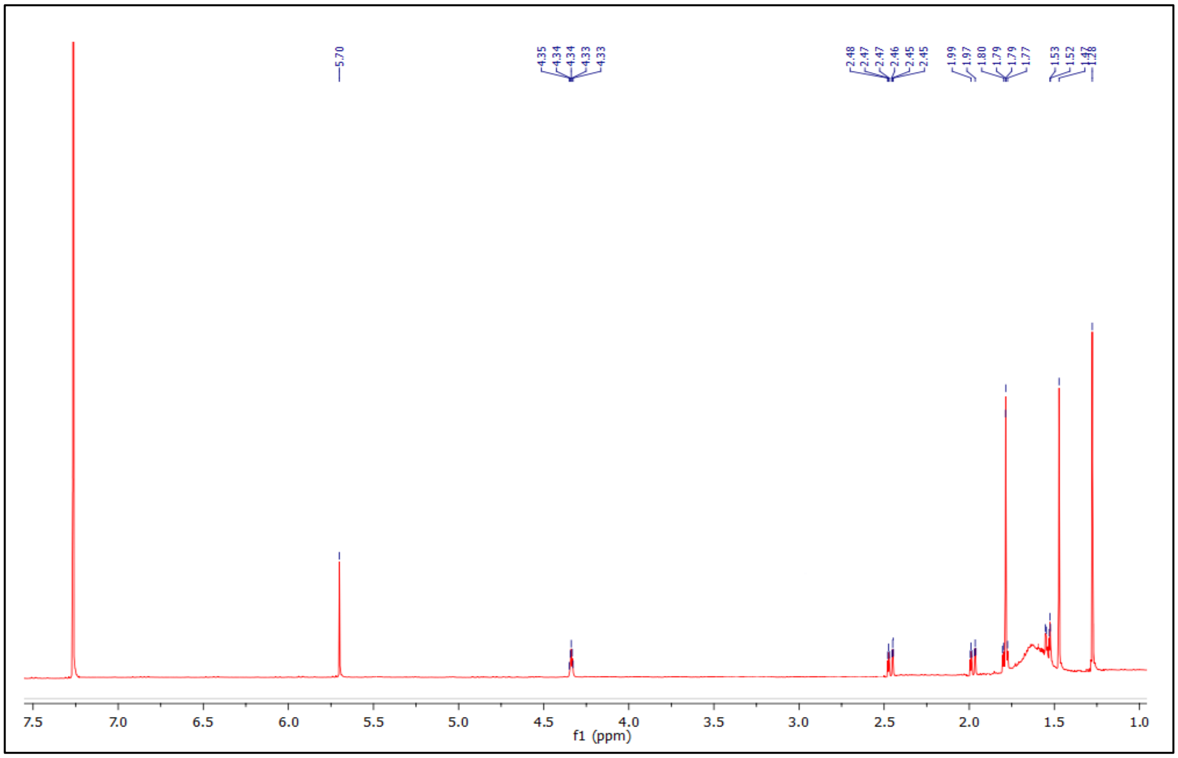

Figure S12. Expansion of ^{1}H NMR spectrum of magnificine B (**2**) ($CDCl_3$).

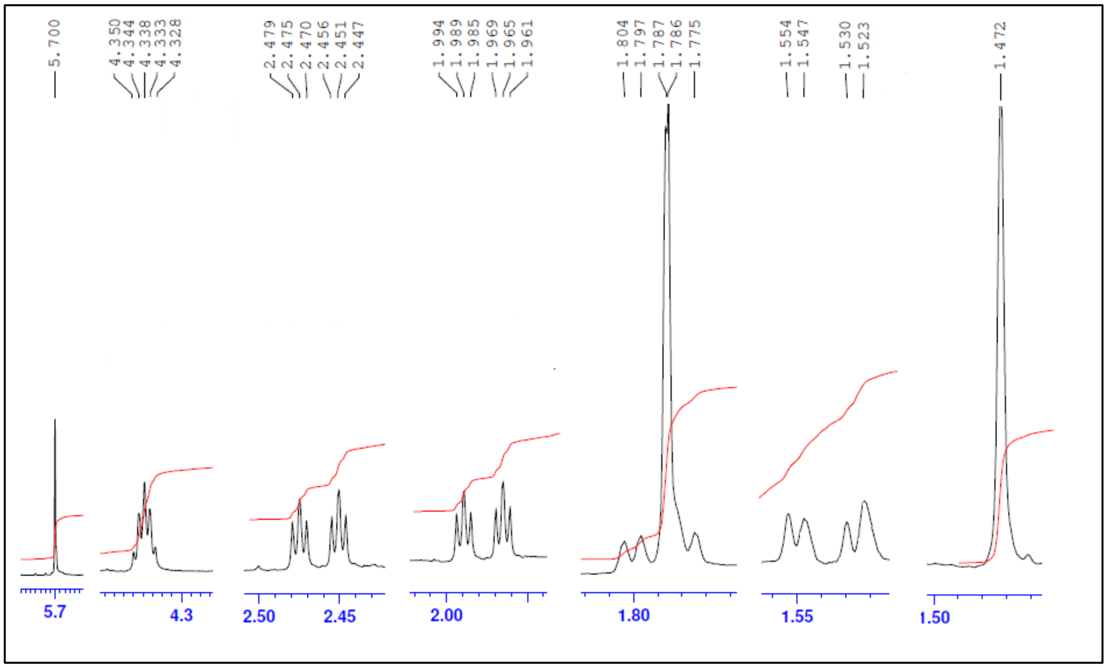

Figure S13. ^{13}C NMR spectrum of magnificine B (**2**) ($CDCl_3$).

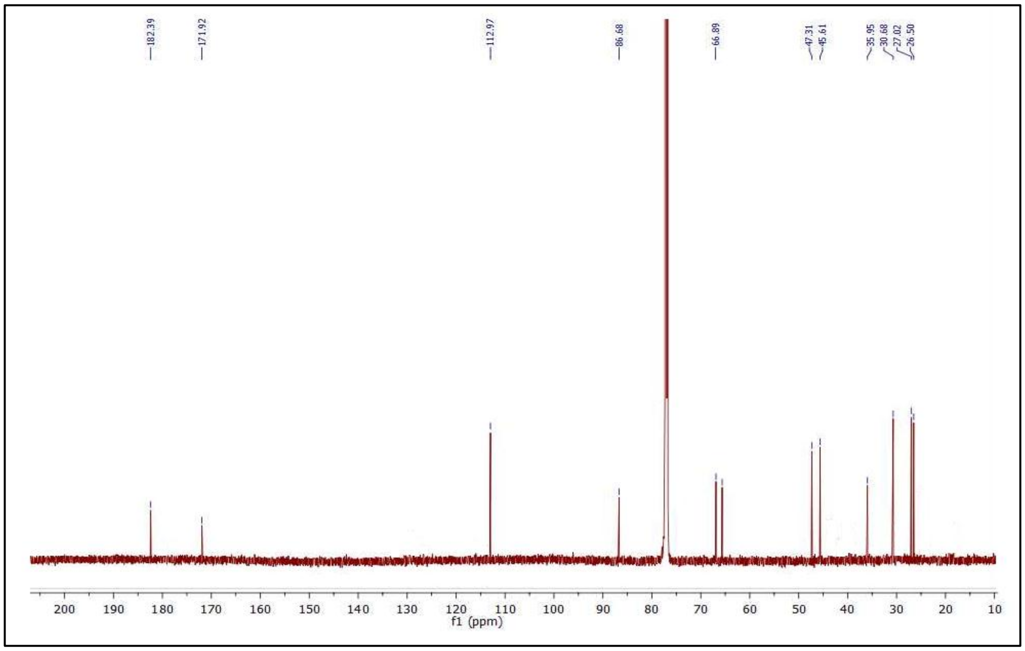

Figure S14. DEPT spectrum of magnificine B (**2**) ($CDCl_3$).

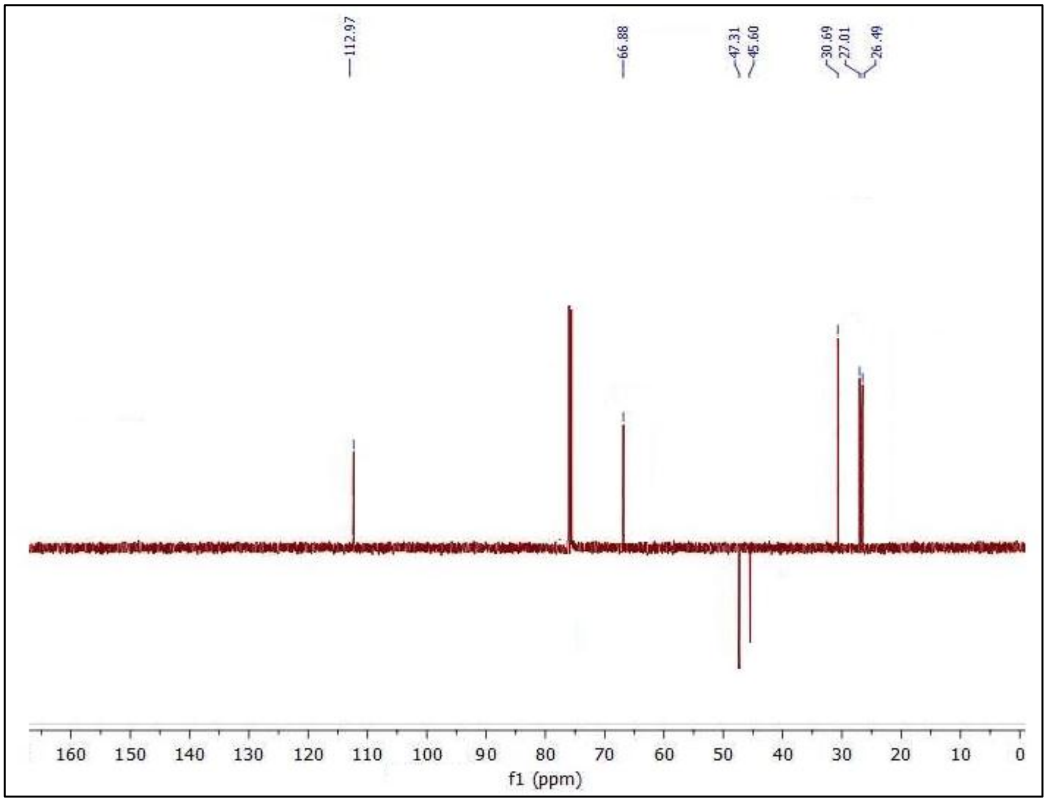

Figure S15. ^{1}H-^{1}H COSY spectrum of magnificine B (**2**) ($CDCl_3$).

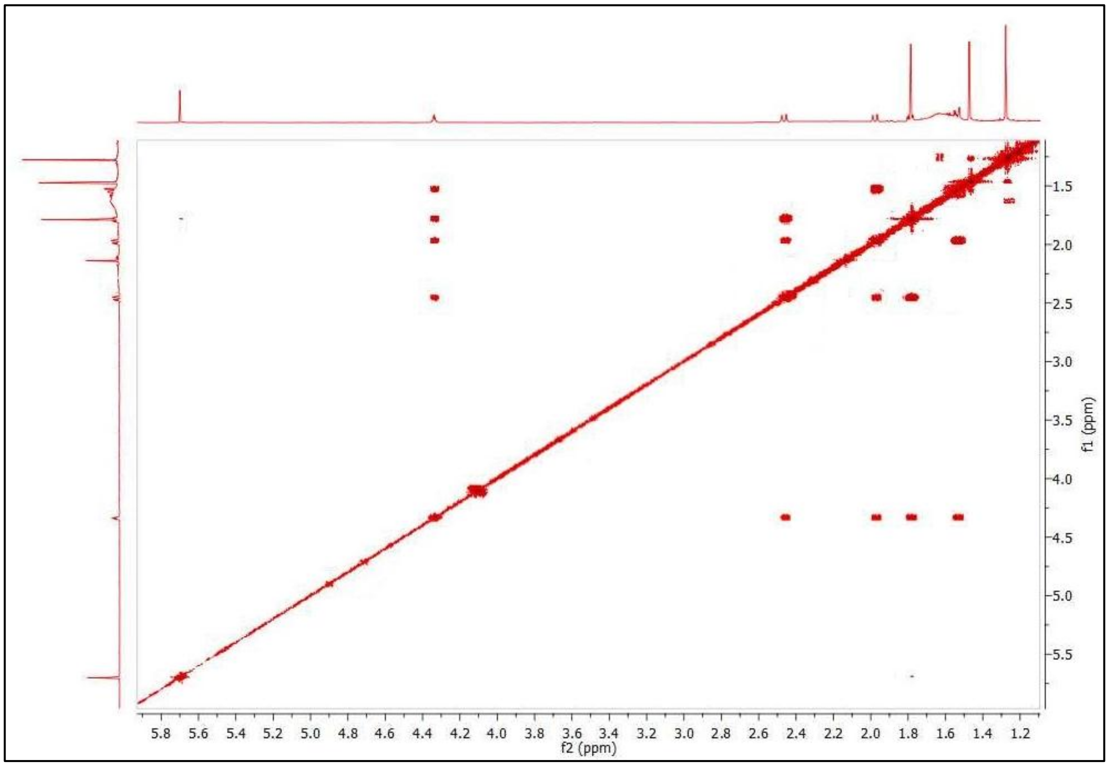

Figure S16. Multiplicity-edited HSQC spectrum of magnificine B (**2**) ($CDCl_3$).

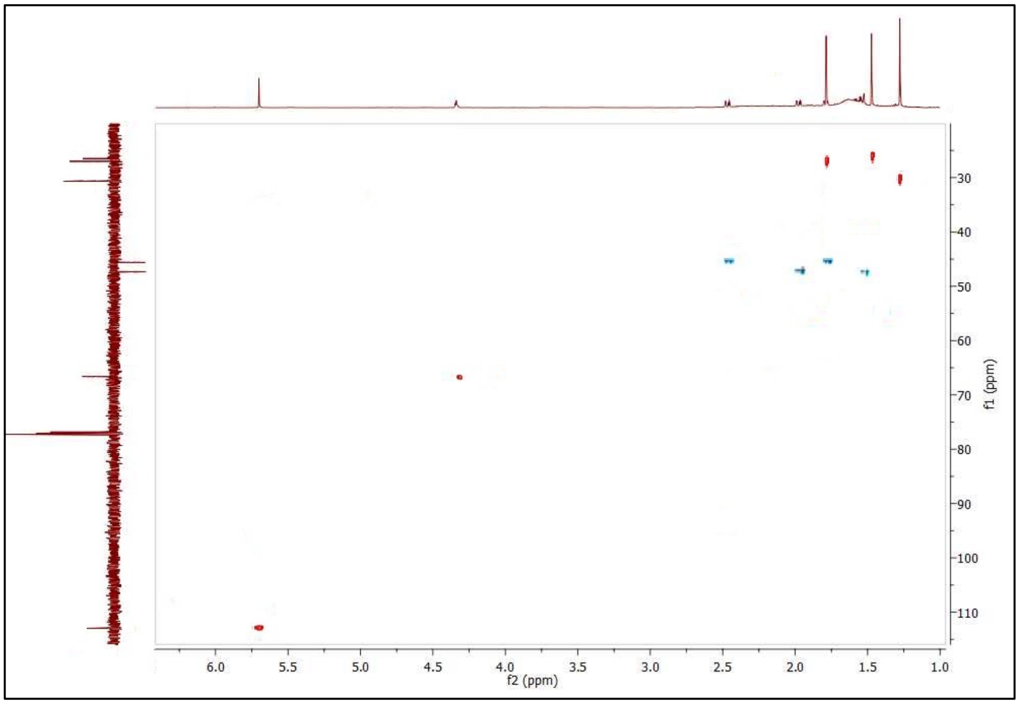

Figure S17. ^{1}H-^{13}C HMBC spectrum of magnificine B (**2**) ($CDCl_3$).

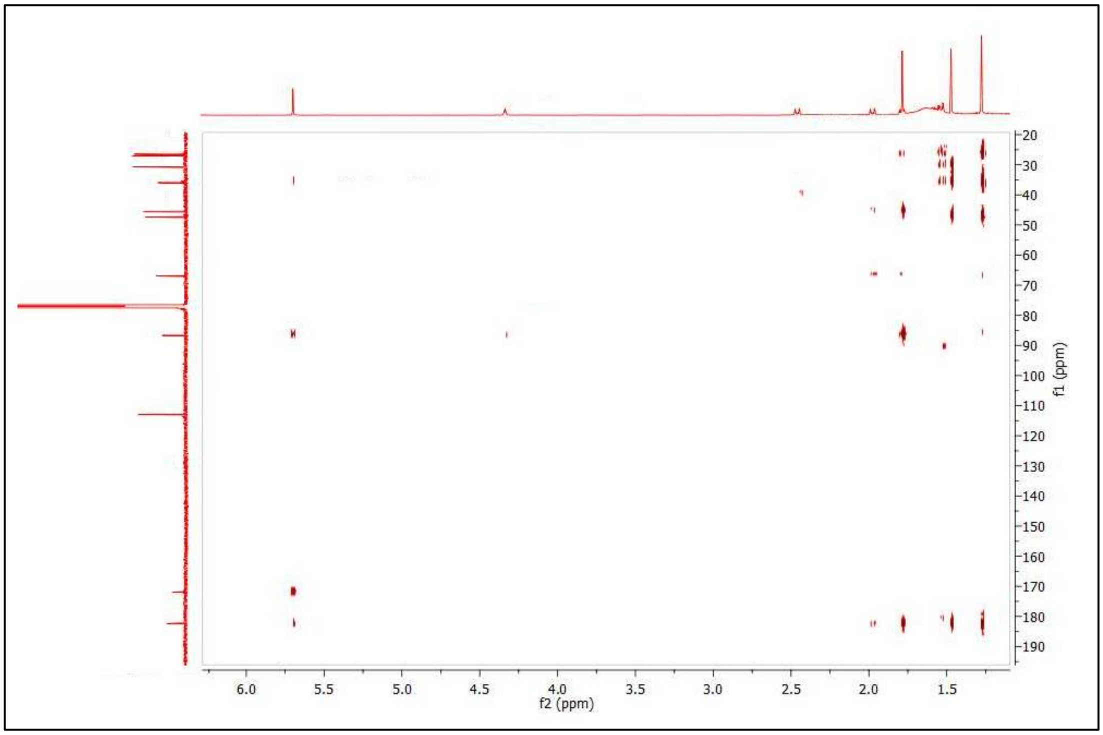

Figure S18. ^{1}H-^{1}H NOESY spectrum of magnificine B (**2**) ($CDCl_3$).

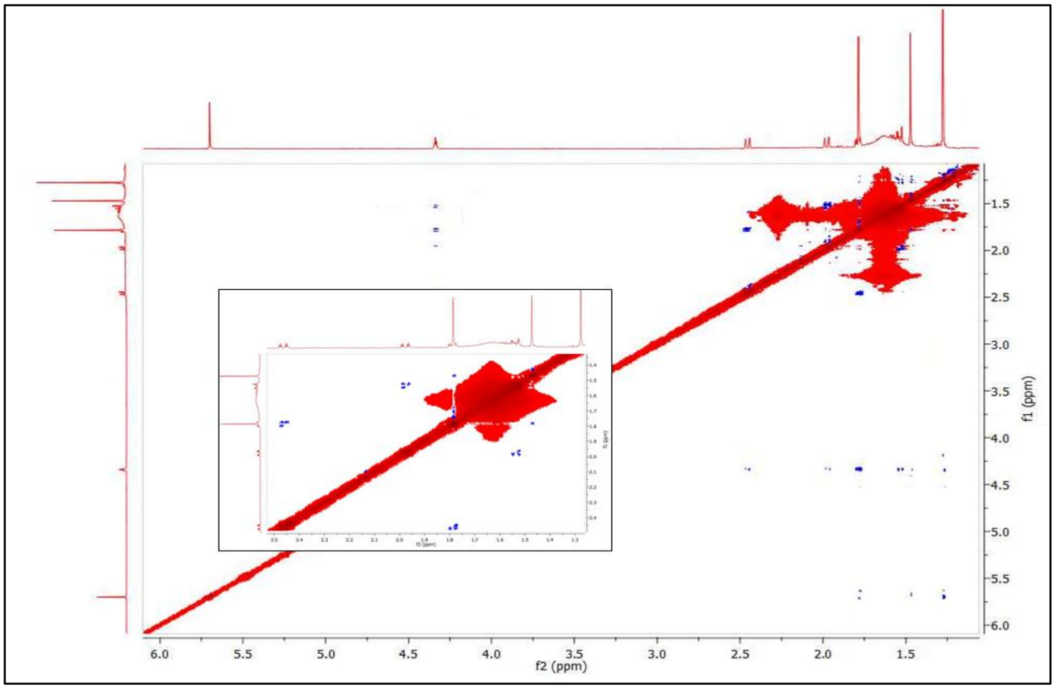

Figure S19. HRESIMS spectrum of magnificine B (**2**).

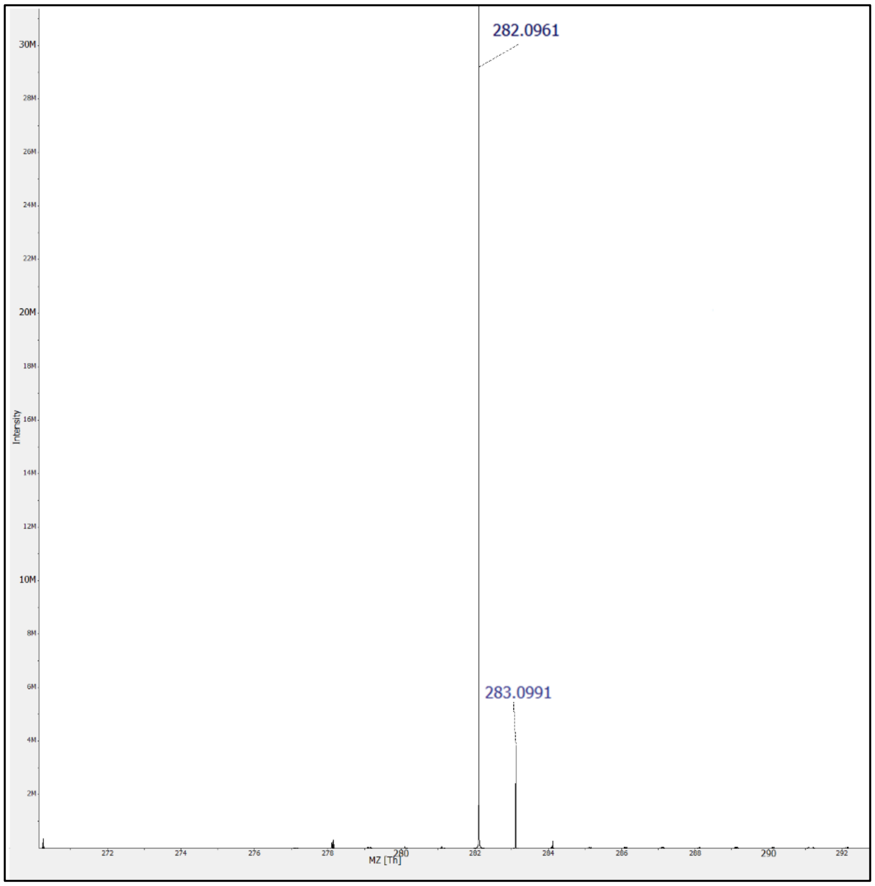

Figure S20. ^{1}H NMR spectrum of (±)-negombaionone (**3**) ($CDCl_3$).

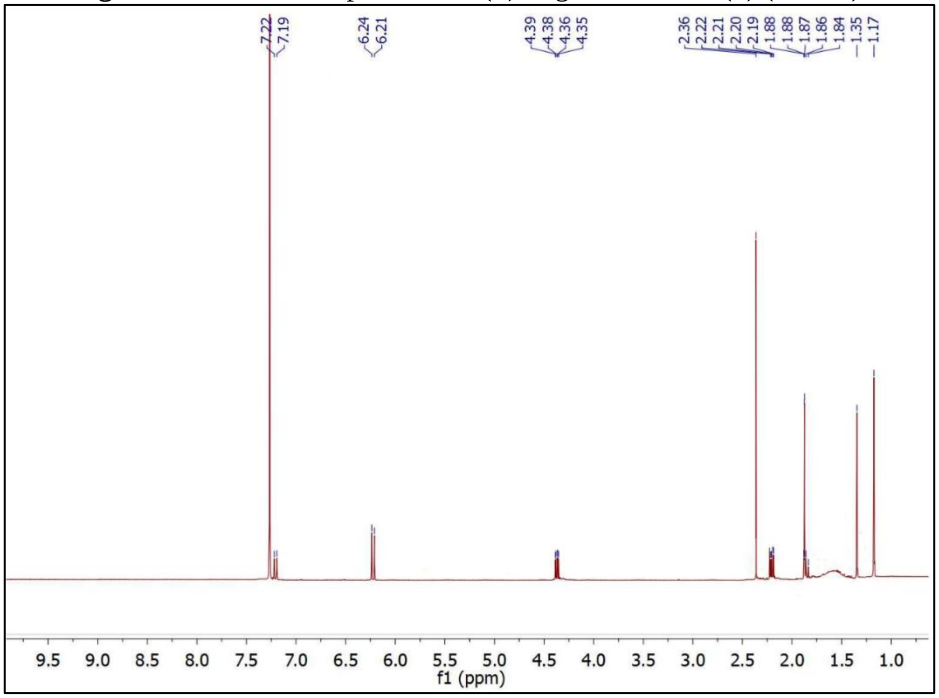

Figure S21. ^{13}C NMR spectrum of (±)-negombaionone (**3**) ($CDCl_3$).

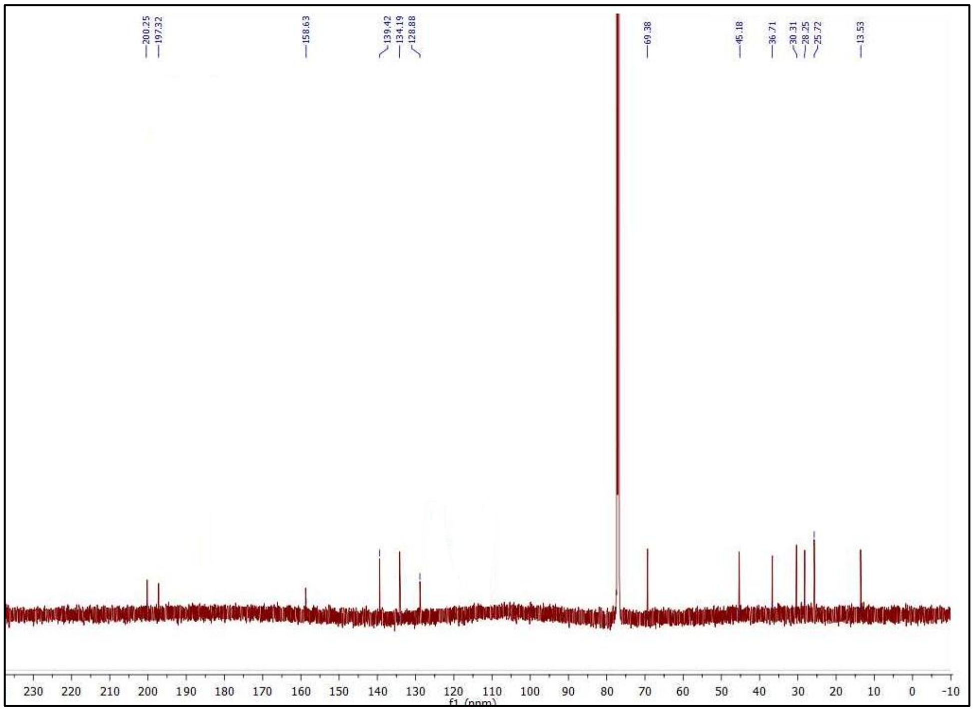

Figure S22. DEPT spectrum of (±)-negombaionone (**3**) ($CDCl_3$).

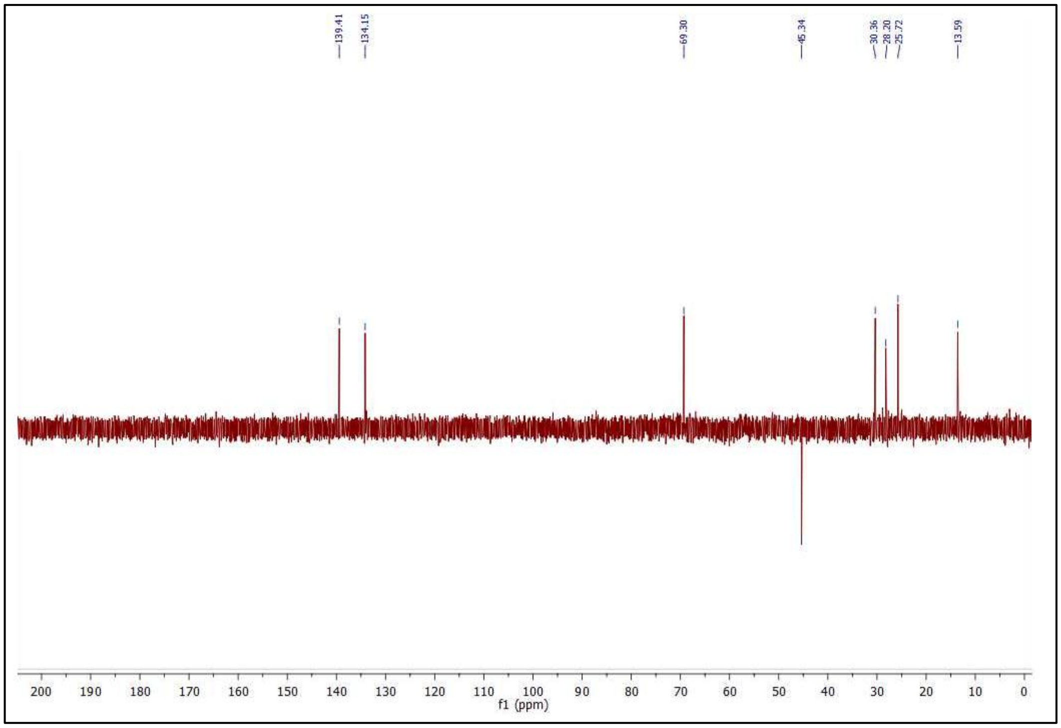

Figure S23. ^{1}H-^{1}H COSY spectrum of (±)-negombaionone (**3**) ($CDCl_3$).

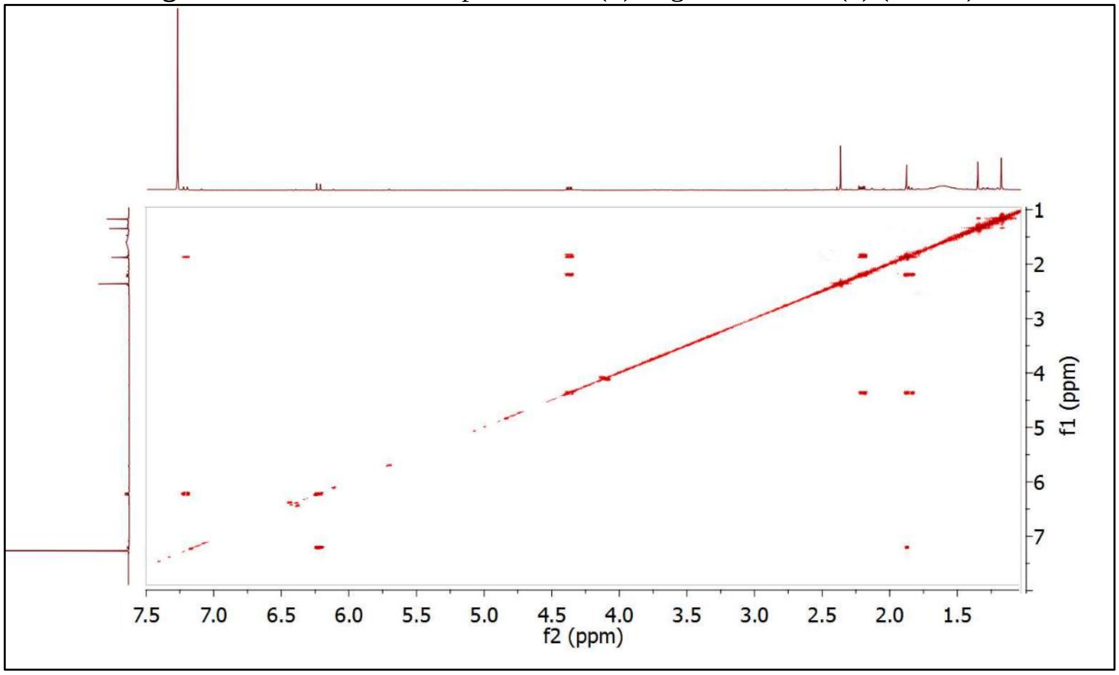

Figure S24. Multiplicity-edited HSQC spectrum of (±)-negombaionone (**3**) ($CDCl_3$).

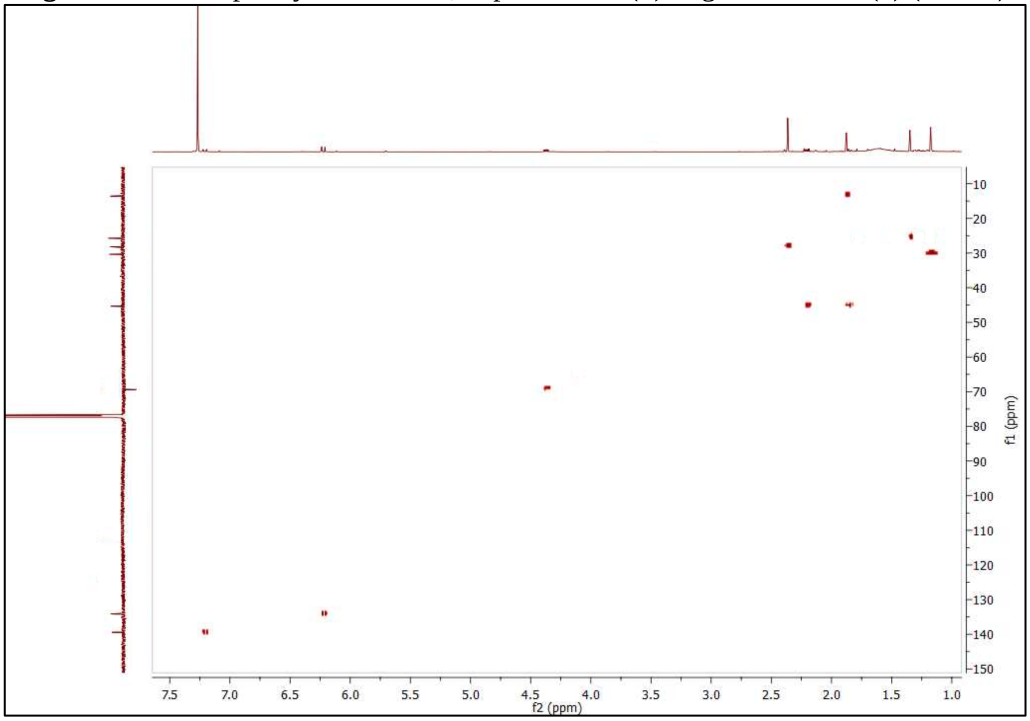

Figure S25. ^{1}H-^{13}C HMBC spectrum of (±)-negombaionone (**3**) ($CDCl_3$).

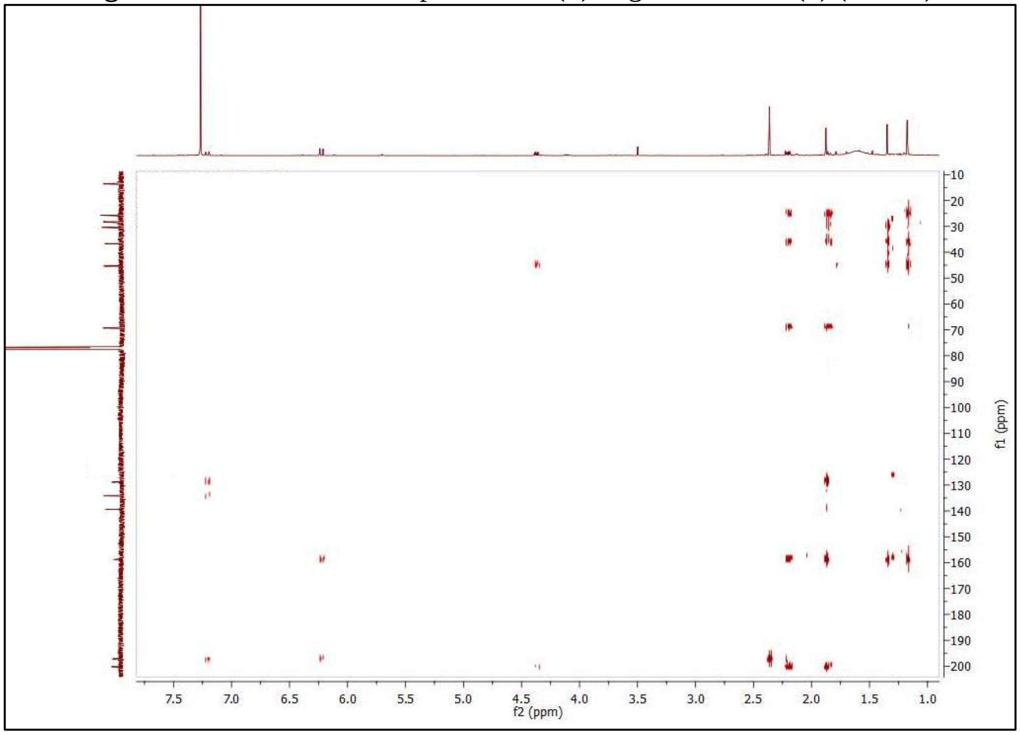

Figure S26. HRESIMS spectrum of (±)-negombaionone (**3**).

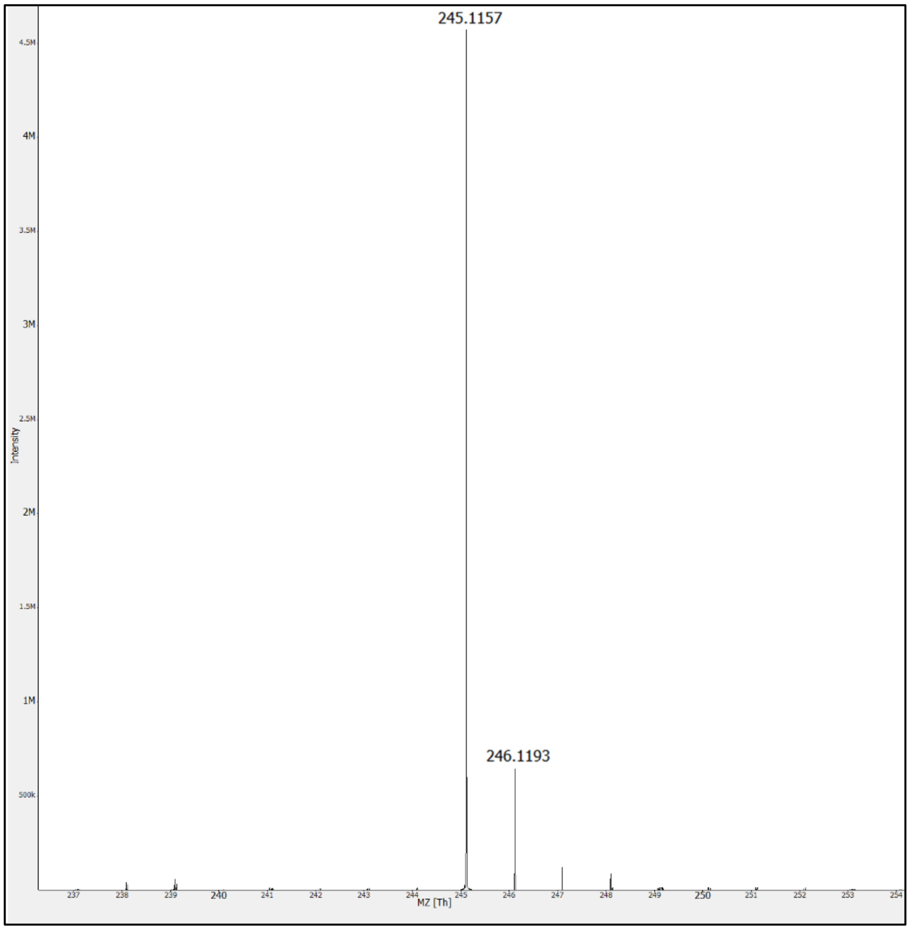

Figure S27. ^{1}H NMR spectrum of latrunculin B (**4**) ($CDCl_3$).

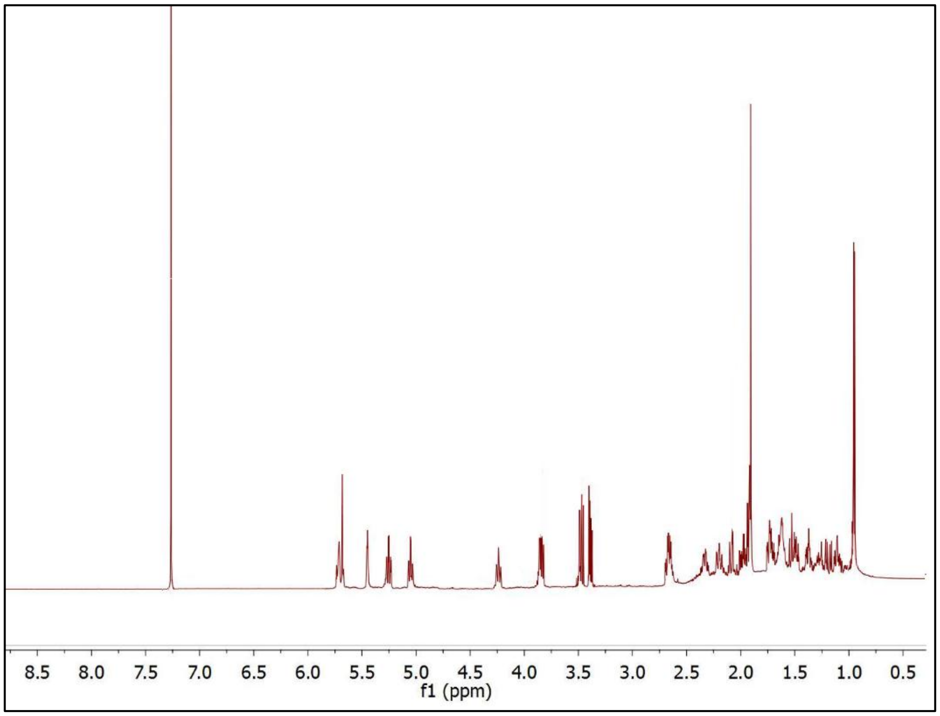

Figure S28. ^{13}C NMR spectrum of latrunculin B (**4**) ($CDCl_3$).

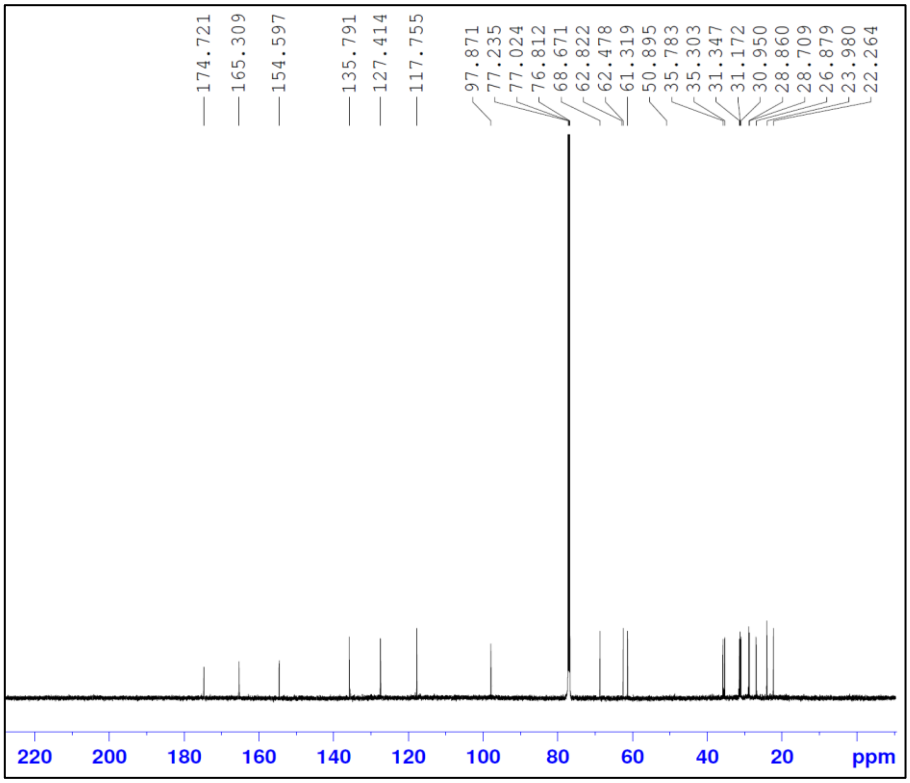

Figure S29. ^{1}H NMR spectrum of 16-epilatrunculin B (**5**) ($CDCl_3$).

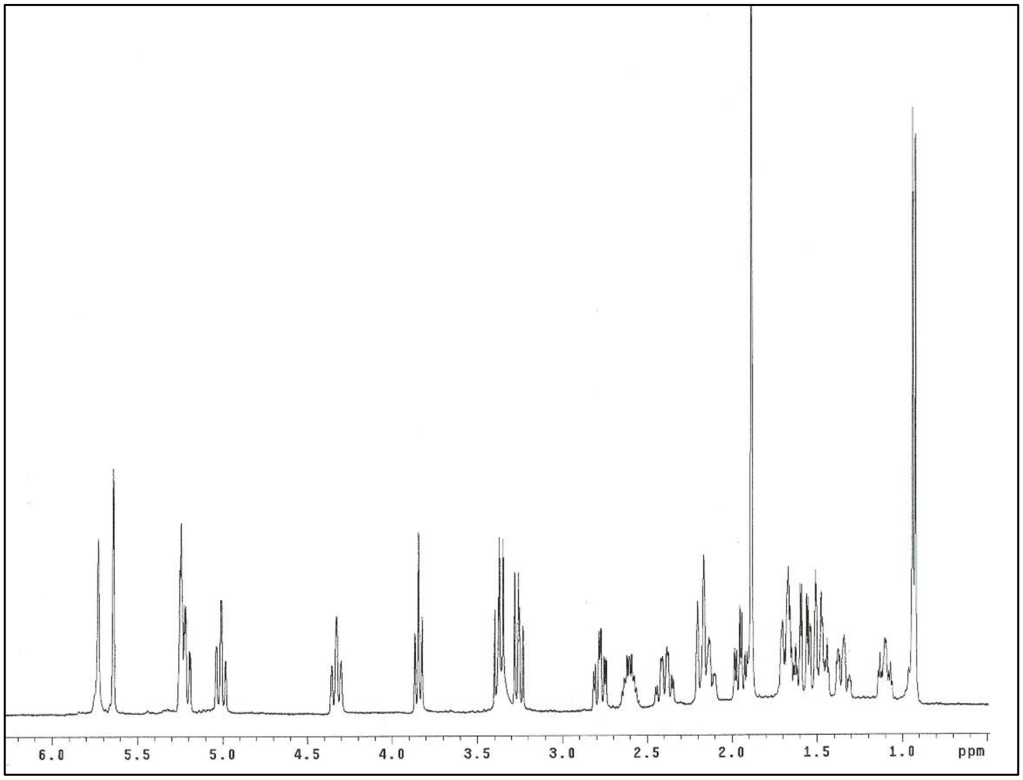

Figure S30. ^{13}C NMR spectrum of 16-epilatrunculin B (**5**) ($CDCl_3$).

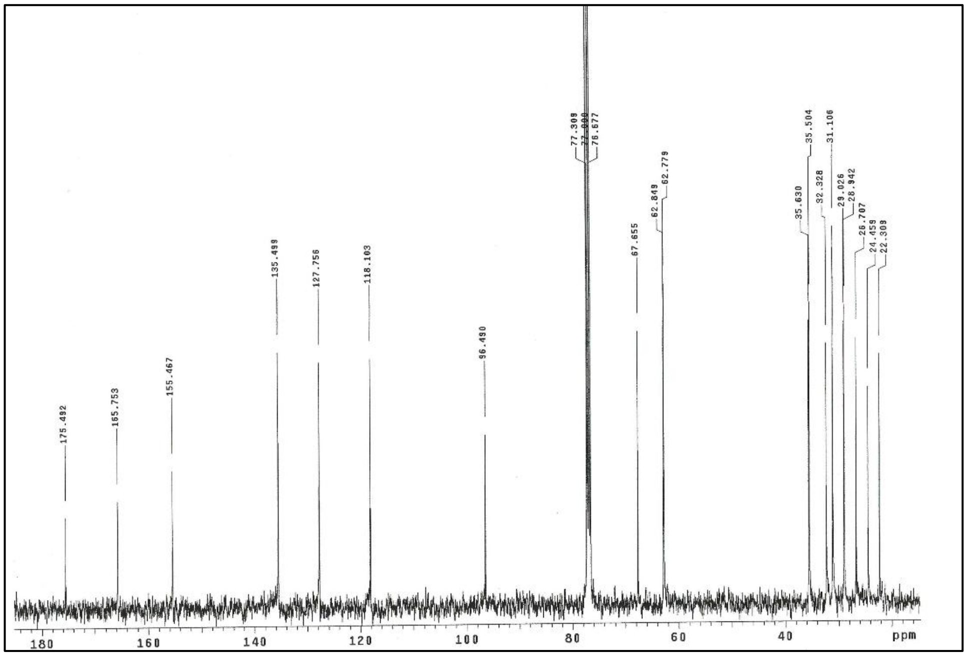

Supplement: Supplementary file 1 [file marinedrugs-19-00214-s001.pdf]
